# Supplementary material for: A study of the associations between social isolation and loneliness with sex-specific cancer risk in the UK Biobank
Source: Commun Med (Lond). 2026 Mar 2;6:200. doi: 10.1038/s43856-026-01429-5 (PMC13066002; doi:10.1038/s43856-026-01429-5)
Supplement: Supplementary file 2 — Supplemental Information [file 43856_2026_1429_MOESM2_ESM.pdf]

Supplemental Online Content

Supplementary Tables 3

eTable1: Competing Risk Proportions by Category. 3

eTable2: Overestimation of Cancer Incidence by Kaplan-Meier Estimates Compared to Cumulative Incidence Functions. 4

eTable3. The Distribution and the ICD-10 Codes of Individual Cancers. 5

eTable4. Baseline Characteristics of the Study Population of Mediation (Hormone). 6

eTable5. Baseline Characteristics of the Study Population of Mediation (Inflammatory Markers). 7

eTable6. Definition of Variables From the UK Biobank Data. 8

eTable7. Matrix of All the Covariates. 10

eTable8. Variance Inflation Factors and Tolerance for Covariates. 11

eTable9. Baseline Characteristics of the Study Population Stratified by Sex (Baseline in 2006.10). 12

eTable10. Cancer Incidence and Excess Incidence in Relation to SI Across Different Demographic Groups. 13

eTable11. Cancer Incidence and Excess Incidence in Relation to Loneliness Across Different Demographic Groups. . 14

eTable12. Indirect Effect (95% Confidence Intervals) and Proportion Mediated (95% Confidence Intervals) of Individual Cancers Associated with Hormone and Menopause Status in Female. 15

eTable13. Indirect Effect (95% Confidence Intervals) and Proportion Mediated (95% Confidence Intervals) of Individual Cancers Associated with Inflammatory Markers in Female Participants. 16

eTable14. Indirect Effect (95% Confidence Intervals) and Proportion Mediated (95% Confidence Intervals) of Individual Cancers Associated with Inflammatory Markers in Male Participants. 17

eTable15. Baseline Characteristics of the Study Population Stratified by Exposures in Sensitive Analysis (Baseline in 2006.10). 18

eTable16: Overall Comparison of Included vs Excluded Participants 19

eTable17: Comparison of Included vs Subgroup Excluded Due to Missing Covariates or Exposures 20

eTable18: Logistic Regression for Missing Data (NA_any) 21

eTable19. Stability of Social Isolation and Loneliness Across Instances Subset with Repeated Assessments. 22

eTable20. Proportion and Trend of Social Isolation and Loneliness by Instance. 23

Supplementary Figures 24

eFigure1. Associations of SI., Loneliness with Organ-Specific Cancer Risk by Cancer Historical Types in Females (Model3). 24

eFigure2. Associations of SI, Loneliness with Organ-Specific Cancer Risk by Cancer Historical Types in Males (Model3). 25

eFigure3. Log-Log Plot of SI and Loneliness. 26

eFigure4. Cumulative Incidence of Cancer by Social Isolation and Loneliness, Accounting for Competing Risks of Death, Over Up to 14 Years of Follow-up. 27

eFigure5. Percentage of Excess Risk Mediated by Covariates for the Association of Loneliness or Social Isolation with Cancer Incidence, in the Overall Population and Stratified by Sex. 28

eFigure6. Associations of SI, Loneliness with Organ-Specific Cancer Risks in Female Participants (Model 1 and Model 2). 29

eFigure7. Associations of SI, Loneliness with Organ-Specific Cancer Risks in Male Participants (Model 1 and Model 2). 30

eFigure8. Associations of SI and Loneliness with Organ-Specific Cancer Risk by Cancer Type and Sex (Model3). 31

eFigure9. Mediation Effects of Hormone, Menopause Status and Inflammatory Markers on Cancer Risk in Participants Exposed to SI. 32

eFigure10. Mediation Effects of Hormone and Menopause Status on Specific Cancer Risk in Female Participants with SI Exposure. 34

eFigure11. Directed Acyclic Graph. 35

eFigure12. Cumulative Incidence of Cancer by Social Isolation and Loneliness, Accounting for Competing Risks of Death, Over Up to 14 Years of Follow-up (Sensitive Analysis). 36

eFigure13. Separate and Joint Association of SI and Loneliness with Long-term Risk of Cancer in Sensitive Analysis.37
eFigure14. Cancer Risk Associated with SI and Loneliness, Stratified by Demographic and Lifestyle Factors in Sensitive Analysis. 38
eFigure15. Associations of SI, Loneliness with Cancer Risk in Male Participants by Cancer Types (Sensitive Analysis).39
eFigure16. Associations of SI, Loneliness with Cancer Risk in Female Participants by Cancer Types (Sensitive Analysis). 40

Supplementary Tables

eTable1: Competing Risk Proportions by Category.

Category	Total Events	CR Events	CR Proportion
**Overall**	50983	12880	25.26%
**Male**	28658	8252	28.79%
**Female**	22325	4628	20.73%
**0-49**	4997	888	17.77%
**50-59**	13681	2712	19.82%
**60 and older**	32305	9280	28.73%

Competing Risk Proportions for Cancer Incidence in Overall, Sex, and Age Group Strata. Proportions calculated as competing events (non-cancer deaths) divided by total events (cancer + competing). N=354,537. Abbreviations: CR, Competing Risk; N, Number.

eTable2: Overestimation of Cancer Incidence by Kaplan-Meier Estimates Compared to Cumulative Incidence Functions.

Group	CIF3yr	KM3yr	Overestimation 3yr	CIF5yr	KM5yr	Overestimation 5yr	CIF10yr	KM10yr	Overestimation 10yr	Death CIF3yr	Death CIF5yr	Death CIF10yr
**Non-isolated**	0.0164	0.0164	0.2	0.0348	0.0349	0.3	0.0956	0.0964	0.9	0.0021	0.0048	0.0188
**Isolated**	0.0214	0.0215	0.5	0.0429	0.0432	0.7	0.1125	0.1149	2.1	0.0053	0.0124	0.046
**Non-lonely**	0.0166	0.0167	0.2	0.0352	0.0353	0.3	0.0966	0.0974	0.9	0.0022	0.005	0.0197
**Lonely**	0.0182	0.0182	0.2	0.0362	0.0364	0.6	0.0975	0.0992	1.6	0.0046	0.0106	0.0349

Comparison of KM and CIF Estimates for Cancer Incidence, with Overestimation and Death CIF, by Exposure Group. Estimates at 3, 5, and 10 years; overestimation = [(KM incidence - CIF cancer) / CIF cancer] × 100. Incidence = 1 - survival for KM. N=354,537. Abbreviations: CIF, Cumulative Incidence Functions; KM, Kaplan-Meier, SI, Social Isolation, YR, Year, N, Number.

eTable3. The Distribution and the ICD-10 Codes of Individual Cancers.

Cancer Name	ICD-10 Code	Total Cases	Male Cases	Female cases	Case Per 10000 Person Years
**Compare**		317 858	144311 (45.4%)	173547 (54.6%)	858.7127045
**Anus Cancer**	**C21**	159	57 (35.8%)	102 (64.2%)	0.4295482
**Biliary duct cancer**	**C23, C24**	215	101 (47%)	114 (53%)	0.5808356
**Bladder cancer**	**C67**	823	639 (77.6%)	184 (22.4%)	2.2233845
**Brain cancer**	**C71**	551	307 (55.7%)	244 (44.3%)	1.4885600
**Breast cancer**	**C50**	6823	52 (0.8%)	6771 (99.2%)	18.4327492
**Colorectal cancer**	**C18, C19, and C20**	4218	2375 (56.3%)	1843 (43.7%)	11.3951833
**Esophageal cancer**	**C15**	734	534 (72.8%)	200 (27.2%)	1.9829456
**Female genitalia cancer**	**C51**	214		214 (100%)	0.5781340
**Kidney cancer**	**C64, C65**	1056	670 (63.4%)	386 (36.6%)	2.8528482
**Laryngeal cancer**	**C32**	121	111 (91.7%)	10 (8.3%)	0.3268889
**Leukemia**	**C91, C92, C93, C94, C95**	946	574 (60.7%)	372 (39.3%)	2.5556765
**Liver cancer**	**C22**	458	300 (65.5%)	158 (34.5%)	1.2373148
**Lung cancer**	**C34**	2751	1401 (50.9%)	1350 (49.1%)	7.4319937
**Lymphoma**	**C81, C82, C83, C84, C85, C86, C88**	1760	945 (53.7%)	815 (46.3%)	4.7547470
**Melanoma**	**C43**	2244	1138 (50.7%)	1106 (49.3%)	6.0623024
**Mesothelioma**	**C45**	291	239 (82.1%)	52 (17.9%)	0.7861542
**Mouth cancer**	**C03, C04, C05, C06**	153	96 (62.7%)	57 (37.3%)	0.4133388
**Multiple myeloma**	**C90.0**	694	402 (57.9%)	292 (42.1%)	1.8748832
**Ovarian cancer**	**C56**	703		703 (100%)	1.8991972
**Pancreatic cancer**	**C25**	953	511 (53.6%)	442 (46.4%)	2.5745874
**Prostate cancer**	**C61**	8446	8446 (100%)		22.8173823
**Small intestine cancer**	**C17**	171	82 (48%)	89 (52%)	0.4619669
**Soft tissue cancer**	**C46, C47, C48, C49**	289	122 (42.2%)	167 (57.8%)	0.7807511
**Stomach cancer**	**C16**	502	340 (67.7%)	162 (32.3%)	1.3561835
**Thyroid cancer**	**C73**	301	81 (26.9%)	220 (73.1%)	0.8131698
**Tongue cancer**	**C01, C02**	222	142 (64%)	80 (36%)	0.5997465
**Tonsil cancer**	**C09**	185	137 (74.1%)	48 (25.9%)	0.4997887
**Uterine cancer**	**C54, C55**	1090		1090 (100%)	2.9447012
**Other cancer**		1191	670 (56.3%)	521 (43.7%)	3.2175589

This table presents the distribution of cancer cases from the UK Biobank, including total cases, cases by sex, and incidence rates per 10,000 person-years, categorized by cancer type and their corresponding ICD-10 codes. Columns include cancer name, ICD-10 code(s), total cases, male and female case counts with percentages, and case rates per 10,000 person-years. The "Compare" column is used for reference comparisons across cancer types. Abbreviation: ICD-10, International Classification of Diseases, 10th Revision.

eTable4. Baseline Characteristics of the Study Population of Mediation (Hormone).

Variable Label	Label	Overall,N = 190,576	No social isolation, N = 179,166	Social isolation, N = 11,410	P-Value
**Had Menopause**					8.81e-81
	**Had hysterestomy,n (%)**	21,199 (11%)	19,694 (11%)	1,505 (13%)	
	**Not menopause,n (%)**	47,830 (25%)	45,804 (26%)	2,026 (18%)	
	**Others,n (%)**	8,190 (4%)	7,734 (4%)	456 (4%)	
	**Had menopause,n (%)**	113,357 (59%)	105,934 (59%)	7,423 (65%)	
	**Unknown**		0	0	
**Sex Hormone-Binding Globulin (nmol/L)**		62.39 ± 31.01	62.43 ± 30.85	61.77 ± 33.41	2.20e-08
	**Unknown**	29,401	27,623	1,778	
**Estradiol (pmol/L)**		535.62 ± 459.02	537.20 ± 461.41	504.13 ± 407.41	1.55e-04
	**Unknown**	147,145	137,817	9,328	
**Ever Used Hormone Replacement Therapy**	**Used hormone replacement therapy**	72,419 (38%)	772,535 (43%)	4,224(37%)	3.18e-37
	**Never used hormone replacement therapy**	118,157 (62%)	10,061 (56%)	7,076(62%)	
	**Others**	419 (0%)	32 (0%)	387 (0%)	
**Age at Menarche (years)**		12.97 ± 1.61	12.97 ± 1.60	12.94 ± 1.71	
	**Unknown**	4,504	4,143	361	

Baseline variates were presented as means ± standard error or median (interquartile range) for continuous variables and frequency (percentages) for categorical variables. Continuous variables were assessed for statistical differences using two-sample T-tests, ANOVA tests, or Mann–Whitney U tests. Categorical variables were evaluated for differences using the $\chi 2$ test. Abbreviations: ANOVA, Analysis of Variance; χ^2, Chi-Squared; SI, Social Isolation.

eTable5. Baseline Characteristics of the Study Population of Mediation (Inflammatory Markers).

Variable Label	Label	Overall,N = 354,537	No social isolation, N = 333,896	Social isolation, N = 20,641	P-Value
**Lymphocytes (×10^9/L)**		1.95 ± 0.81	1.95 ± 0.80	2.00 ± 0.86	4.41e-19
	**Unknown**	13,525	12,669	856	
**Monocytes (×10^9/L)**		0.48 ± 0.21	0.47 ± 0.21	0.49 ± 0.23	1.27e-09
	**Unknown**	13,525	12,669	856	
**Neutrophils (×10^9/L)**		4.21 ± 1.39	4.19 ± 1.38	4.54 ± 1.59	9.26e-204
	**Unknown**	13,525	12,669	856	
**Platelets (×10^9/L)**		252.33 ± 59.28	252.02 ± 59.03	257.31 ± 62.85	6.18e-30
	**Unknown**	12,886	12,073	813	
**White Blood Cells (×10^9/L)**		6.85 ± 1.83	6.83 ± 1.82	7.25 ± 2.08	2.78e-181
	**Unknown**	12,886	12,073	813	
**C-Reactive Protein (mg/L)**		2.61 ± 4.39	2.61 ± 4.40	2.59 ± 4.28	3.76e-01
	**Unknown**	23,998	22,628	1,370	
**Lymphocytes to Monocytes Ratio**		4.60 ± 3.52	4.60 ± 3.46	4.71 ± 4.35	5.43e-02
	**Unknown**	13,629	12,764	865	
**Neutrophils to Lymphocytes Ratio**		2.34 ± 1.25	2.34 ± 1.24	2.50 ± 1.38	9.93e-73
	**Unknown**	13,530	12,674	856	
**Platelets to Lymphocytes Ratio**		141.31 ± 71.08	141.24 ± 71.35	142.45 ± 66.48	5.80e-01
	**Unknown**	13,534	12,678	856	
**Systemic Inflammation**		594.19 ± 371.22	591.00 ± 366.70	646.06 ± 434.93	3.50e-94
	**Unknown**	13,534	12,678	856	

Baseline variates were presented as means ± standard error or median (interquartile range) for continuous variables. Continuous variables were assessed for statistical differences using two-sample T-tests or Mann–Whitney U tests. Abbreviations: SI, social isolation.

eTable6. Definition of Variables From the UK Biobank Data.

Characteristic	Source	Notes	UK Biobank field	Coding notes
**Outcome**				
**Age at first cancer event**	Cancer Registry, Death Registry	Date of cancer diagnosis	Data-Field 40005	
**Specific cancer type**	Cancer Registry	Histology of cancer tumour;Type of cancer: ICD10	Data-Field 40011; Data-Field 40006	
**Age at loss-to-follow up**	Death Registry	Date of death	Data-Field 40000	
**Exposure**				
**SI**	Baseline	Number in household; Frequency of friend/family visits; Leisure/social activities	Data-Field 709; Data-Field 1031; Data-Field 6160	
**Loneliness**	Baseline	Able to confide; Loneliness, isolation	Data-Field 2110; Data-Field 2020	
**Covariates**				
**Age**	Baseline	Age when attended assessment centre	Data-Field 21003	Continuous variable, years
**Sex**	Baseline		Data-Field 31	Male, Female
**Ethnicity**	Baseline	Ethnic background	Data-Field 21000	White, Non-White
**Smoking status**	Baseline	Smoking status	Data-Field 20116	Never, Previous, Current
**Alcohol status**	Baseline	Alcohol drinker status	Data-Field 20117	Never, Previous, Current
**Assessment center**	Baseline	UK Biobank assessment centre	Data-Field 54	England; Scotland; Wales
**Sun exposure time**	Baseline	Time spend outdoors in summer; Time spent outdoors in winter	Data-Field 1050; Data-Field 1060	Continuous variable, Hours/day
**Diet score**	Baseline	Cooked vegetable intake; Salad / raw vegetable intake; Fresh fruit intake; Dried fruit intake; Oily fish intake;Non-oily fish intake; Processed meat intake; Beef intake; Lamb/mutton intake; Pork intake	Data-Field 1289; Data-Field 1299; Data-Field 1309; Data-Field 1319; Data-Field 1329; Data-Field 1339; Data-Field 1349; Data-Field 1369; Data-Field 1379; Data-Field 1389	Evaluated by a healthy diet score based on the intake of vegetable, fish, unprocessed red meat, and processed meat, continuous variable
**Healthy sleep pattern**	Baseline	Sleep duration; Morning/evening person (chronotype); Sleeplessness/ insomnia; Snoring; Daytime dozing / sleeping	Data-Field1160; Data-Field 1180; Data-Field 1200; Data-Field 1210; Data-Field 1220	Evaluated by a healthy sleep score based on sleep traits including insomnia, sleep duration, chronotype, daytime sleepiness, and snoring [range 0-5], continuous variable
**BMI**	Baseline	Body mass index (BMI)	Data-Field 21001	Continuous variable, Kg/m^2
**MAP**	Baseline	automated reading of systolic blood pressure and diastolic blood pressure calculated as [SBP + (2 × DBP)] / 3)	Data-Field 4080; Data-Field 93; Data-Field 4079; Data-Field 94	Continuous variable, mmHg
**Grip strength**	Baseline	Mean of left and right hand grip strength	Data-Field 46; Data-Field 47	Continuous variable, kg
**Family history of cancer**	Baseline	Illnesses of father; Illnesses of mother; Illnesses of siblings	Data-Field 20107; Data-Field 20110; Data-Field 20111	yes or no
**Depress mood**	Baseline	Frequency of depressed mood in last 2 weeks	Data-Field 2050	Based on the frequency of depressed mood in the previous 2 weeks from the Patient Health Questionnaire
**Townsend deprivation index**	Baseline	Townsend deprivation index at recruitment	Data-Field 22189	Townsend Deprivation Score was calculated based on the area of residence at baseline, indicating socioeconomic status, with higher scores indicating greater deprivation.
**Stratification Variables**				
**Household income**	Baseline	Average total household income before tax	Data-Field 738	High: ≥ £52 000, Medium: £18 000-£51 999, and Low: < £18 000
**Educational levels**	Baseline	Qualifications	Data-Field 6138	College/university degree, Non-College/university degree
**Employment status**	Baseline	Current employment status	Data-Field 6142	Employed, Retired, Others
**Mediators**				
**Had menopause**	Baseline	Had menopause	Data-Field 2724	Yes or No
**SHBG level**	Baseline	Blood biochemistry	Data-Field 30830	Continuous, nmol/L
**Oestradiol level**	Baseline	Blood biochemistry	Data-Field 30800	Continuous, pmol/L
**C-reactive protein**	Baseline	Blood biochemistry	Data-Field 30710	Continuous, mg/L
**HRT history**	Baseline	Ever used HRT	Data-Field 2814	Yes or No
**Menarche age**	Baseline	Age when periods started (menarche)	Data-Field 2714	Continuous, years
**Lym**	Baseline	Lymphocyte count	Data-Field 30120	Continuous, 10^9 cells/Litre
**Mono**	Baseline	Monocyte count	Data-Field 30130	Continuous, 10^9 cells/Litre
**Neu**	Baseline	Neutrophill count	Data-Field 30140	Continuous, 10^9 cells/Litre
**PLT**	Baseline	Platelet count	Data-Field 30080	Continuous, 10^9 cells/Litre
**WBC**	Baseline	White blood cell (leukocyte) count	Data-Field 30000	Continuous, 10^9 cells/Litre
**Systemic inflammation**	Baseline			

Variables used in the study are listed in the table, including outcomes, exposures, covariates, stratification variables, and mediators, sourced from the UK Biobank dataset. Columns detail the variable characteristics, data source, notes on measurement, UK Biobank field identifiers, and coding methods. Abbreviations: BMI, Body Mass Index; HRT, Hormone-Replacement Therapy; MAP, Mean Arterial Pressure; SI, Social Isolation; Lym, Lymphocytes; Mono, Monocytes; Neu, Neutrophils; PLT, Platelets; SHBG, Sex Hormone-Binding Globulin; WBC, White Blood Cells.

eTable7. Matrix of All the Covariates.

Age	Sex	Race	BMI	UK Center	Income	Education	Employment	Smoking status	Alcohol status	Depress mood	TDI	Family cancer	Overall health rating	Hand grip	Day exposure	Diet score	Sleep score	MAP
0.02****																		
0.11****	0																	
0.04****	0.07****	0																
-0.01****	0**	0.05****	0.02****															
0.17****	-0.08****	-0.04****	0.03****	-0.01****														
0.18****	-0.09****	0.03****	0.06****	-0.01****	0.19****													
0.62****	-0.05****	0.06****	0.02****	-0.01****	0.18****	0.18****												
0.15****	0.03****	0.07****	0.07****	-0.02****	0	0	0.08****											
0.02****	-0.04****	-0.14****	0.03****	0	0.04****	0.05****	0.05****	-0.03****										
0.04****	0**	0.02****	-0.03****	0*	0.04****	0.01****	0.01****	0.02****	-0.02****									
-0.09****	0.01****	-0.19****	0.07****	-0.02****	0.05****	0.05****	-0.04****	-0.08****	0.11****	-0.03****								
0.04****	0****	0.07****	0.01****	0	0**	0**	0.01****	0.01****	-0.01****	-0.01****	-0.02****							
-0.02****	-0.05****	0.08****	-0.21****	0.02****	-0.1****	-0.12****	-0.09****	0.04****	-0.12****	0.05****	-0.18****	0**						
-0.19****	0.73****	0.04****	0.05****	0.03****	-0.13****	-0.15****	-0.19****	0.01****	-0.09****	0****	-0.05****	0****	0.09****					
0.13****	0.18****	0	0.05****	0.01****	0.12****	0.14****	0.14****	0**	0.01****	0.01****	0.05****	0	-0.01****	0.13****				
0.09****	-0.19****	-0.04****	-0.05****	-0.02****	0.02****	-0.01****	0.06****	0.06****	0.02****	0***	-0.01****	0**	0.09****	-0.14****	0.04****			
-0.07****	0.05****	0.03****	-0.08****	-0.01****	-0.04****	-0.08****	-0.08****	0	-0.06****	0.07****	-0.09****	-0.02****	0.22****	0.1****	-0.01****	0		
0.2****	0.16****	0.01****	0.24****	0.03****	0.05****	0.05****	0.11****	0.05****	-0.03****	0.01****	-0.03****	0	-0.06****	0.13****	0.08****	-0.03****	-0.01****	

Pearson correlation coefficients were calculated for covariates using complete cases from the UK Biobank dataset. Significance: **** $p < 0.0001$; *** $p < 0.001$; ** $p < 0.01$; * $p < 0.05$; 0 indicates non-significant or near-zero correlation. Abbreviations: MAP, Mean Arterial Pressure; TDI, Townsend Deprivation Index; BMI, Body Mass Index.

eTable8. Variance Inflation Factors and Tolerance for Covariates.

Variable	GVIF	Df	Adjusted_VIF	Tolerance
**Age**	1.984	1	1.984	0.5041
**Sex**	2.472	1	2.472	0.4046
**Race**	1.109	1	1.109	0.9016
**MAP**	1.147	1	1.147	0.8719
**BMI**	1.154	1	1.154	0.8668
**UK Center**	1.012	2	1.006	0.9942
**Income**	1.456	3	1.133	0.8823
**Education**	1.134	1	1.134	0.8816
**Employment**	2.005	2	1.416	0.7062
**Smoking status**	1.125	2	1.061	0.9428
**Alcohol status**	1.099	2	1.049	0.9537
**Depress mood**	1.078	2	1.038	0.9630
**Townsend deprivation index**	1.155	1	1.155	0.8656
**Family cancer**	1.007	1	1.007	0.9933
**Overall health rating**	1.250	1	1.250	0.8001
**Hand grip**	2.532	1	2.532	0.3949
**Day exposure**	1.119	1	1.119	0.8934
**Diet score**	1.070	1	1.070	0.9342
**Sleep score**	1.077	1	1.077	0.9283

VIF and tolerance values were computed in a multivariable linear regression framework treating each covariate as the outcome in turn, with GVIF for categorical variables adjusted as GVIF^(1/(2*Df)), where Df is degrees of freedom. Tolerance = 1/Adjusted VIF. Higher VIF indicates greater multicollinearity. Abbreviation: VIF, Variance Inflation Factors;GVIF; Generalized Variance Inflation Factors;; Df, Degrees of Freedom; MAP, Mean Arterial Pressure; BMI, Body Mass Index.

eTable9. Baseline Characteristics of the Study Population Stratified by Sex (Baseline in 2006.10).

Variable Label	Label	Overall, N = 354,537	Male, N = 190,576	Female, N = 163,961	P-Value
Social isolated,n (%)		20,641 (6%)	11,410 (6%)	9,231 (6%)	2.11e-142
Lonely,n (%)		15,942 (4%)	7,713 (4%)	8,229 (5%)	7.24e-521
Age, years		56.34 ± 8.06	56.05 ± 8.00	56.67 ± 8.11	3.67e-112
Ethnicity,White,n (%)		340,360 (96%)	182,802 (96%)	157,558 (96%)	8.12e-03
Assessment Center					3.10e-02
	English,n (%)	325,924 (92%)	175,038 (92%)	150,886 (92%)	
	Scotland,n (%)	12,992 (4%)	7,130 (4%)	5,862 (4%)	
	Wales,n (%)	15,621 (4%)	8,408 (4%)	7,213 (4%)	
Employment					1.07e-289
	Employed,n (%)	211,397 (60%)	108,921 (57%)	102,476 (63%)	
	Others,n (%)	28,422 (8%)	17,482 (9%)	10,940 (7%)	
	Retired,n (%)	114,718 (32%)	64,173 (34%)	50,545 (31%)	
College/university degree, n (%)		178,566 (50%)	87,241 (46%)	91,325 (56%)	3.91e-756
Income					3.28e-1021
	High,n (%)	87,548 (25%)	41,436 (22%)	46,112 (28%)	
	Low,n (%)	64,489 (18%)	36,945 (19%)	27,544 (17%)	
	Medium,n (%)	162,895 (46%)	85,621 (45%)	77,274 (47%)	
	Unreported,n (%)	39,605 (11%)	26,574 (14%)	13,031 (8%)	
Smoking status					7.35e-803
	Current,n (%)	35,339 (10%)	16,177 (8%)	19,162 (12%)	
	Never,n (%)	195,266 (55%)	113,806 (60%)	81,460 (50%)	
	Previous,n (%)	123,932 (35%)	60,593 (32%)	63,339 (39%)	
Alcohol status					5.01e-413
	Current,n (%)	329,778 (93%)	174,635 (92%)	155,143 (95%)	
	Never,n (%)	13,025 (4%)	9,414 (5%)	3,611 (2%)	
	Previous,n (%)	11,734 (3%)	6,527 (3%)	5,207 (3%)	
Family cancer history, n (%)		111,083 (31%)	59,887 (31%)	51,196 (31%)	2.01e-01
Townsend Deprivation Score		-1.48 ± 2.97	-1.48 ± 2.93	-1.48 ± 3.01	2.13e-03
Sun exposure time, hours/day		2.83 ± 1.89	2.52 ± 1.52	3.19 ± 2.19	1.88e-2293
Diet Score		2.18 ± 1.21	2.39 ± 1.18	1.94 ± 1.20	1.50e-2743
Sleep Score		2.39 ± 0.75	2.35 ± 0.77	2.42 ± 0.72	1.37e-185
Depress mood					1.22e-95
	High,n (%)	15,551 (4%)	9,302 (5%)	6,249 (4%)	
	Low,n (%)	328,107 (93%)	174,739 (92%)	153,368 (94%)	
	Unreported,n (%)	10,879 (3%)	6,535 (3%)	4,344 (3%)	
BMI (kg/m2)		27.26 ± 4.86	26.86 ± 5.20	27.72 ± 4.40	4.64e-612
MAP(mmHg)		101.23 ± 12.47	99.23 ± 12.53	103.55 ± 12.00	3.44e-2349
Grip strength, kg		31.00 ± 11.00	23.52 ± 6.21	39.69 ± 8.74	1.65e-53823
Overall Health Rating		2.90 ± 0.73	2.94 ± 0.71	2.86 ± 0.75	1.09e-222

Baseline variates were presented as means ± standard error or median (interquartile range) for continuous variables and frequency (percentages) for categorical variables. Continuous variables were assessed for statistical differences using two-sample T-tests, ANOVA tests, or Mann–Whitney U tests. Categorical variables were evaluated for differences using the $\chi 2$ test. Abbreviations: ANOVA, Analysis of Variance; BMI, Body Mass Index; MAP, Mean Arterial Pressure; χ^2, Chi-Squared; SI, Social Isolation.

eTable10. Cancer Incidence and Excess Incidence in Relation to SI Across Different Demographic Groups.

group	exposure	N incident	cases	Person years	Incident per 10000 person years	Five Year CIF	IRR	Excess Incidence
**Male**		163,961	36,910	1,690,925.87	218	3.97% (3.87%, 4.06%)		
	**Unexposed**	154,730	33,689	1,599,125.71	211	3.94% (3.85%, 4.04%)	1 [Reference]	0 [Reference]
	**Exposed**	9,231	3,221	91,800.16	351	4.34% (3.92%, 4.76%)	1.67 (1.61 to 1.73)	140.2 (139.53 to 140.86)
**Female**		190,576	26,953	2,009,415.31	134	3.15% (3.07%, 3.23%)		
	**Unexposed**	179,166	24,562	1,891,762.64	130	3.08% (3%, 3.16%)	1 [Reference]	0 [Reference]
	**Exposed**	11,410	2,391	117,652.67	203	4.27% (3.9%, 4.65%)	1.57 (1.5 to 1.63)	73.39 (72.8 to 73.98)
**0-49**		84,942	5,885	884,674.00	67	1.43% (1.35%, 1.51%)		
	**Unexposed**	81,087	5,442	845,288.65	64	1.42% (1.34%, 1.5%)	1 [Reference]	0 [Reference]
	**Exposed**	3,855	443	39,385.35	112	1.56% (1.17%, 1.96%)	1.75 (1.59 to 1.92)	48.1 (47.09 to 49.11)
**50-59**		120,239	16,393	1,279,080.73	128	2.77% (2.68%, 2.86%)		
	**Unexposed**	112,840	14,837	1,202,773.25	123	2.71% (2.62%, 2.81%)	1 [Reference]	0 [Reference]
	**Exposed**	7,399	1,556	76,307.48	204	3.63% (3.2%, 4.06%)	1.65 (1.57 to 1.74)	80.56 (79.82 to 81.29)
**59-**		149,356	41,585	1,536,586.44	271	5.32% (5.21%, 5.44%)		
	**Unexposed**	139,969	37,972	1,442,826.45	263	5.28% (5.16%, 5.4%)	1 [Reference]	0 [Reference]
	**Exposed**	9,387	3,613	93,759.99	385	5.95% (5.47%, 6.43%)	1.46 (1.42 to 1.52)	122.17 (121.51 to 122.83)
**High Income**		87,548	9,872	937,236.10	105	2.56% (2.45%, 2.66%)		
	**Unexposed**	85,970	9,646	920,787.01	105	2.56% (2.46%, 2.67%)	1 [Reference]	0 [Reference]
	**Exposed**	1,578	226	16,449.09	137	2.31% (1.57%, 3.06%)	1.31 (1.15 to 1.5)	32.64 (31.09 to 34.18)
**Low Income**		64,489	17,743	653,600.19	271	4.61% (4.45%, 4.78%)		
	**Unexposed**	55,433	14,590	563,134.81	259	4.56% (4.38%, 4.73%)	1 [Reference]	0 [Reference]
	**Exposed**	9,056	3,153	90,465.38	349	4.97% (4.52%, 5.42%)	1.35 (1.29 to 1.4)	89.45 (88.74 to 90.15)
**Medium Income**		162,895	27,853	1,702,330.83	164	3.55% (3.46%, 3.64%)		
	**Unexposed**	154,942	26,352	1,620,342.62	163	3.54% (3.44%, 3.63%)	1 [Reference]	0 [Reference]
	**Exposed**	7,953	1,501	81,988.20	183	3.9% (3.47%, 4.33%)	1.13 (1.07 to 1.19)	20.44 (19.74 to 21.14)
**Unreported Income**		39,605	8,395	407,174.05	206	3.81% (3.62%, 4%)		
	**Unexposed**	37,551	7,663	386,623.91	198	3.78% (3.58%, 3.97%)	1 [Reference]	0 [Reference]
	**Exposed**	2,054	732	20,550.14	356	4.49% (3.59%, 5.39%)	1.8 (1.67 to 1.94)	158 (156.6 to 159.4)
**College/university degree**		178,566	27,625	1,884,411.42	147	3.16% (3.08%, 3.24%)		
	**Unexposed**	169,628	25,759	1,792,465.25	144	3.13% (3.05%, 3.21%)	1 [Reference]	0 [Reference]
	**Exposed**	8,938	1,866	91,946.17	203	3.65% (3.26%, 4.04%)	1.41 (1.35 to 1.48)	59.24 (58.57 to 59.9)
**Not college degree**		175,971	36,238	1,815,929.76	200	3.91% (3.81%, 4%)		
	**Unexposed**	164,268	32,492	1,698,423.11	191	3.84% (3.75%, 3.94%)	1 [Reference]	0 [Reference]
	**Exposed**	11,703	3,746	117,506.65	319	4.8% (4.41%, 5.19%)	1.67 (1.61 to 1.72)	127.48 (126.89 to 128.07)
**Employed**		211,397	26,118	2,226,848.74	117	2.65% (2.59%, 2.72%)		
	**Unexposed**	200,228	24,253	2,111,367.97	115	2.62% (2.55%, 2.69%)	1 [Reference]	0 [Reference]
	**Exposed**	11,169	1,865	115,480.77	161	3.33% (3%, 3.67%)	1.41 (1.34 to 1.47)	46.63 (46.04 to 47.22)
**Retired**		114,718	31,681	1,182,331.43	268	5.23% (5.1%, 5.36%)		
	**Unexposed**	108,134	29,043	1,117,020.17	260	5.17% (5.04%, 5.31%)	1 [Reference]	0 [Reference]
	**Exposed**	6,584	2,638	65,311.27	404	6.21% (5.63%, 6.8%)	1.55 (1.49 to 1.62)	143.91 (143.12 to 144.7)
**Other Employment**		28,422	6,064	291,161.01	208	3.12% (2.92%, 3.32%)		
	**Unexposed**	25,534	4,955	262,500.22	189	3.06% (2.84%, 3.27%)	1 [Reference]	0 [Reference]
	**Exposed**	2,888	1,109	28,660.79	387	3.69% (3%, 4.39%)	2.05 (1.92 to 2.19)	198.18 (196.96 to 199.4)
**Previous smoker**		123,932	26,235	1,296,153.67	202	4.04% (3.93%, 4.15%)		
	**Unexposed**	116,977	24,140	1,225,534.39	197	4% (3.88%, 4.11%)	1 [Reference]	0 [Reference]
	**Exposed**	6,955	2,095	70,619.29	297	4.71% (4.21%, 5.22%)	1.51 (1.44 to 1.57)	99.69 (98.93 to 100.44)
**Current smoker**		35,339	9,433	356,021.95	265	4.27% (4.06%, 4.49%)		
	**Unexposed**	31,231	7,838	315,559.50	248	4.08% (3.86%, 4.3%)	1 [Reference]	0 [Reference]
	**Exposed**	4,108	1,595	40,462.44	394	5.75% (5.03%, 6.47%)	1.59 (1.5 to 1.67)	145.81 (144.77 to 146.84)
**Never smoker**		195,266	28,195	2,048,165.56	138	3.07% (2.99%, 3.15%)		
	**Unexposed**	185,688	26,273	1,949,794.46	135	3.05% (2.98%, 3.13%)	1 [Reference]	0 [Reference]
	**Exposed**	9,578	1,922	98,371.10	195	3.38% (3.02%, 3.75%)	1.45 (1.38 to 1.52)	60.64 (59.99 to 61.28)
**Current drinker**		329,778	58,167	3,448,030.39	169	3.52% (3.46%, 3.59%)		
	**Unexposed**	312,023	53,667	3,267,105.60	164	3.48% (3.42%, 3.55%)	1 [Reference]	0 [Reference]
	**Exposed**	17,755	4,500	180,924.80	249	4.22% (3.92%, 4.52%)	1.51 (1.47 to 1.56)	84.46 (83.98 to 84.93)
**Previous drinker**		11,734	3,107	119,560.99	260	4.08% (3.72%, 4.44%)		
	**Unexposed**	10,061	2,386	103,333.25	231	3.89% (3.51%, 4.27%)	1 [Reference]	0 [Reference]
	**Exposed**	1,673	721	16,227.74	444	5.22% (4.15%, 6.29%)	1.92 (1.77 to 2.09)	213.4 (211.74 to 215.05)
**Nondrinker**		13,025	2,589	132,749.79	195	3.18% (2.87%, 3.48%)		
	**Unexposed**	11,812	2,198	120,449.51	182	3.07% (2.75%, 3.38%)	1 [Reference]	0 [Reference]
	**Exposed**	1,213	391	12,300.28	318	4.26% (3.11%, 5.4%)	1.74 (1.56 to 1.94)	135.4 (133.54 to 137.25)

Incidence per 10,000 person-years was calculated by dividing the number of cancer cases by the total person-years for each group, then multiplying by 10,000. The 5-year CIF was used to estimate the probability of developing cancer within 5 years. IRRs were calculated by comparing the cancer incidence rate for each group exposed to SI to the reference group; Excess incidence represents the additional cancer cases per 10000 person-years observed in the exposed groups compared to the unexposed groups. Abbreviations: SI, Social Isolation; CI, Confidence Interval; CIF, Cumulative Incidence Function; IRR, Incidence Rate Ratio; N, Number.

eTable11. Cancer Incidence and Excess Incidence in Relation to Loneliness Across Different Demographic Groups.

group	exposure	N incident	cases	Person years	Incident per 10000 person years	Five Year CIF	IRR	Excess Incidence
**Male**		163,961	36,910	1,690,925.868	218	3.97% (3.87%, 4.06%)		
	**Unexposed**	155,732	34,705	1,606,955.941	216	3.97% (3.88%, 4.07%)	1 [Reference]	0 [Reference]
	**Exposed**	8,229	2,205	83,969.927	263	3.81% (3.39%, 4.23%)	1.22 (1.16 to 1.27)	46.63 (45.93 to 47.32)
**Female**		190,576	26,953	2,009,415.310	134	3.15% (3.07%, 3.23%)		
	**Unexposed**	182,863	25,561	1,928,463.442	133	3.14% (3.06%, 3.22%)	1 [Reference]	0 [Reference]
	**Exposed**	7,713	1,392	80,951.869	172	3.42% (3.01%, 3.82%)	1.3 (1.23 to 1.37)	39.41 (38.7 to 40.11)
**0-49**		84,942	5,885	884,674.004	67	1.43% (1.35%, 1.51%)		
	**Unexposed**	80,956	5,545	843,486.441	66	1.45% (1.36%, 1.53%)	1 [Reference]	0 [Reference]
	**Exposed**	3,986	340	41,187.564	83	1.03% (0.71%, 1.34%)	1.26 (1.13 to 1.4)	16.81 (15.82 to 17.8)
**50-59**		120,239	16,393	1,279,080.729	128	2.77% (2.68%, 2.86%)		
	**Unexposed**	114,385	15,272	1,217,548.790	125	2.75% (2.65%, 2.84%)	1 [Reference]	0 [Reference]
	**Exposed**	5,854	1,121	61,531.938	182	3.23% (2.77%, 3.69%)	1.45 (1.37 to 1.54)	56.75 (55.94 to 57.56)
**59-**		149,356	41,585	1,536,586.445	271	5.32% (5.21%, 5.44%)		
	**Unexposed**	143,254	39,449	1,474,384.152	268	5.31% (5.19%, 5.43%)	1 [Reference]	0 [Reference]
	**Exposed**	6,102	2,136	62,202.293	343	5.66% (5.08%, 6.24%)	1.28 (1.23 to 1.34)	75.83 (75.03 to 76.64)
**High Income**		87,548	9,872	937,236.102	105	2.56% (2.45%, 2.66%)		
	**Unexposed**	85,212	9,571	912,408.459	105	2.56% (2.46%, 2.67%)	1 [Reference]	0 [Reference]
	**Exposed**	2,336	301	24,827.642	121	2.29% (1.68%, 2.9%)	1.16 (1.03 to 1.3)	16.34 (15.08 to 17.6)
**Low Income**		64,489	17,743	653,600.194	271	4.61% (4.45%, 4.78%)		
	**Unexposed**	59,569	16,089	604,073.063	266	4.56% (4.39%, 4.73%)	1 [Reference]	0 [Reference]
	**Exposed**	4,920	1,654	49,527.131	334	5.23% (4.61%, 5.86%)	1.25 (1.19 to 1.32)	67.62 (66.7 to 68.53)
**Medium Income**		162,895	27,853	1,702,330.828	164	3.55% (3.46%, 3.64%)		
	**Unexposed**	156,093	26,660	1,631,052.997	163	3.58% (3.48%, 3.67%)	1 [Reference]	0 [Reference]
	**Exposed**	6,802	1,193	71,277.831	167	3.04% (2.63%, 3.45%)	1.02 (0.97 to 1.09)	3.92 (3.17 to 4.67)
**Unreported Income**		39,605	8,395	407,174.055	206	3.81% (3.62%, 4%)		
	**Unexposed**	37,721	7,946	387,884.864	205	3.85% (3.65%, 4.04%)	1 [Reference]	0 [Reference]
	**Exposed**	1,884	449	19,289.191	233	3.14% (2.34%, 3.93%)	1.14 (1.03 to 1.25)	27.92 (26.47 to 29.36)
**College/university degree**		178,566	27,625	1,884,411.422	147	3.16% (3.08%, 3.24%)		
	**Unexposed**	171,749	26,349	1,812,789.920	145	3.17% (3.08%, 3.25%)	1 [Reference]	0 [Reference]
	**Exposed**	6,817	1,276	71,621.502	178	2.9% (2.5%, 3.3%)	1.23 (1.16 to 1.3)	32.81 (32.06 to 33.55)
**Not college degree**		175,971	36,238	1,815,929.756	200	3.91% (3.81%, 4%)		
	**Unexposed**	166,846	33,917	1,722,629.463	197	3.89% (3.8%, 3.99%)	1 [Reference]	0 [Reference]
	**Exposed**	9,125	2,321	93,300.293	249	4.16% (3.74%, 4.57%)	1.26 (1.21 to 1.32)	51.88 (51.22 to 52.53)
**Employed**		211,397	26,118	2,226,848.735	117	2.65% (2.59%, 2.72%)		
	**Unexposed**	202,577	24,893	2,134,146.813	117	2.66% (2.59%, 2.73%)	1 [Reference]	0 [Reference]
	**Exposed**	8,820	1,225	92,701.923	132	2.44% (2.12%, 2.77%)	1.13 (1.07 to 1.2)	15.5 (14.84 to 16.16)
**Retired**		114,718	31,681	1,182,331.433	268	5.23% (5.1%, 5.36%)		
	**Unexposed**	110,138	30,071	1,135,762.313	265	5.21% (5.08%, 5.34%)	1 [Reference]	0 [Reference]
	**Exposed**	4,580	1,610	46,569.121	346	5.82% (5.14%, 6.51%)	1.31 (1.24 to 1.37)	80.96 (80.03 to 81.88)
**Other Employment**		28,422	6,064	291,161.009	208	3.12% (2.92%, 3.32%)		
	**Unexposed**	25,880	5,302	265,510.257	200	3.06% (2.85%, 3.27%)	1 [Reference]	0 [Reference]
	**Exposed**	2,542	762	25,650.752	297	3.71% (2.97%, 4.45%)	1.49 (1.38 to 1.6)	97.38 (96.09 to 98.66)
**Previous smoker**		123,932	26,235	1,296,153.674	202	4.04% (3.93%, 4.15%)		
	**Unexposed**	118,387	24,803	1,238,601.200	200	4.03% (3.92%, 4.15%)	1 [Reference]	0 [Reference]
	**Exposed**	5,545	1,432	57,552.475	249	4.1% (3.58%, 4.62%)	1.24 (1.18 to 1.31)	48.57 (47.73 to 49.4)
**Current smoker**		35,339	9,433	356,021.946	265	4.27% (4.06%, 4.49%)		
	**Unexposed**	32,660	8,557	329,202.866	260	4.23% (4.01%, 4.45%)	1 [Reference]	0 [Reference]
	**Exposed**	2,679	876	26,819.079	327	4.77% (3.96%, 5.59%)	1.26 (1.17 to 1.35)	66.7 (65.46 to 67.95)
**Never smoker**		195,266	28,195	2,048,165.558	138	3.07% (2.99%, 3.15%)		
	**Unexposed**	187,548	26,906	1,967,615.317	137	3.08% (3%, 3.16%)	1 [Reference]	0 [Reference]
	**Exposed**	7,718	1,289	80,550.241	160	2.87% (2.49%, 3.25%)	1.17 (1.11 to 1.24)	23.28 (22.58 to 23.98)
**Current drinker**		329,778	58,167	3,448,030.394	169	3.52% (3.46%, 3.59%)		
	**Unexposed**	315,480	55,035	3,299,718.869	167	3.52% (3.46%, 3.59%)	1 [Reference]	0 [Reference]
	**Exposed**	14,298	3,132	148,311.525	211	3.54% (3.23%, 3.84%)	1.27 (1.22 to 1.31)	44.39 (43.87 to 44.91)
**Previous drinker**		11,734	3,107	119,560.992	260	4.08% (3.72%, 4.44%)		
	**Unexposed**	10,841	2,813	110,556.936	254	4.03% (3.66%, 4.4%)	1 [Reference]	0 [Reference]
	**Exposed**	893	294	9,004.056	327	4.64% (3.26%, 6.03%)	1.28 (1.14 to 1.45)	72.08 (69.93 to 74.23)
**Nondrinker**		13,025	2,589	132,749.793	195	3.18% (2.87%, 3.48%)		
	**Unexposed**	12,274	2,418	125,143.578	193	3.13% (2.82%, 3.44%)	1 [Reference]	0 [Reference]
	**Exposed**	751	171	7,606.215	225	3.94% (2.53%, 5.34%)	1.16 (1 to 1.36)	31.6 (29.28 to 33.91)

Incidence per 10,000 person-years was calculated by dividing the number of cancer cases by the total person-years for each group, then multiplying by 10,000. The 5-year CIF was used to estimate the probability of developing cancer within 5 years. IRRs were calculated by comparing the cancer incidence rate for each group exposed to loneliness to the reference group; Excess incidence represents the additional cancer cases per 10000 person-years observed in the exposed groups compared to the unexposed groups. Abbreviations: CI, Confidence Interval; CIF, Cumulative Incidence Function; IRR, Incidence Rate Ratio; N, Number.

eTable12. Indirect Effect (95% Confidence Intervals) and Proportion Mediated (95% Confidence Intervals) of Individual Cancers Associated with Hormone and Menopause Status in Female.

Mediator	Bladder Cancer	Breast Cancer	Lung Cancer	Ovarian Cancer	Stomach Cancer	Uterine Cancer
**Indirect Effect (95% Confidence Intervals)**						
**Age menarche**	-0.0013 [-0.0042 - 0.0016]	0.0001 [-0.0003 - 0.0005]	0.0004 [-0.0006 - 0.0013]	-0.0002 [-0.0014 - 0.0010]	0.0003 [-0.0022 - 0.0028]	0.0011 [-0.0006 - 0.0028]
**Ever used HRT no**	0.0001 [-0.0008 - 0.0010]	0.0000 [-0.0001 - 0.0002]	-0.0001 [-0.0004 - 0.0003]	0.0005 [-0.0009 - 0.0019]	0.0009 [-0.0018 - 0.0036]	0.0010 [-0.0019 - 0.0038]
**Ever used HRT others**	0.0035 [-0.6074 - 0.6144]	-0.0000 [-0.0001 - 0.0000]	-0.0000 [-0.0010 - 0.0009]	-0.0002 [-0.0010 - 0.0006]	0.0035 [-0.6114 - 0.6185]	0.0036 [-0.2404 - 0.2475]
**Ever used HRT yes**	0.0001 [-0.0007 - 0.0009]	0.0000 [-0.0001 - 0.0002]	-0.0000 [-0.0003 - 0.0003]	0.0005 [-0.0010 - 0.0020]	0.0008 [-0.0018 - 0.0033]	0.0008 [-0.0019 - 0.0036]
**Had menopause hysterectomy**	0.0002 [-0.0011 - 0.0016]	-0.0002 [-0.0008 - 0.0004]	0.0002 [-0.0004 - 0.0009]	-0.0015 [-0.0048 - 0.0019]	-0.0011 [-0.0038 - 0.0016]	-0.0107 [-0.0344 - 0.0131]
**Had menopause no**	-0.0004 [-0.0027 - 0.0019]	0.0005 [-0.0004 - 0.0015]	-0.0012 [-0.0036 - 0.0013]	0.0005 [-0.0009 - 0.0020]	0.0007 [-0.0019 - 0.0032]	0.0014 [-0.0020 - 0.0049]
**Had menopause others**	0.0005 [-0.0020 - 0.0031]	-0.0003 [-0.0008 - 0.0003]	-0.0002 [-0.0009 - 0.0006]	-0.0001 [-0.0009 - 0.0006]	0.0007 [-0.0021 - 0.0036]	-0.0004 [-0.0015 - 0.0006]
**Had menopause yes**	-0.0000 [-0.0016 - 0.0015]	0.0001 [-0.0003 - 0.0006]	0.0000 [-0.0005 - 0.0006]	-0.0010 [-0.0029 - 0.0010]	-0.0010 [-0.0035 - 0.0016]	-0.0020 [-0.0068 - 0.0028]
**Oestradiol**	-0.0008 [-0.0073 - 0.0057]	0.0003 [-0.0010 - 0.0017]	0.0007 [-0.0039 - 0.0054]	-0.0001 [-0.0028 - 0.0027]	-0.0032 [-0.0134 - 0.0070]	0.0008 [-0.0028 - 0.0043]
**SHBG**	0.0072 [-0.0031 - 0.0176]	-0.0031 [-0.0051 - -0.0010]	-0.0004 [-0.0044 - 0.0035]	0.0050 [-0.0007 - 0.0107]	-0.0116 [-0.0256 - 0.0023]	-0.0169 [-0.0248 - -0.0091]
**Proportion Mediated (95% Confidence Intervals)**						
**Age menarche**	-0.0021 [-0.0069 - 0.0027]	0.0008 [-0.0029 - 0.0044]	0.0013 [-0.0020 - 0.0046]	-0.0004 [-0.0029 - 0.0021]	0.0004 [-0.0032 - 0.0041]	0.0035 [-0.0025 - 0.0095]
**Ever used HRT no**	0.0001 [-0.0013 - 0.0016]	0.0002 [-0.0013 - 0.0017]	-0.0002 [-0.0015 - 0.0011]	0.0011 [-0.0019 - 0.0040]	0.0013 [-0.0027 - 0.0053]	0.0031 [-0.0064 - 0.0126]
**Ever used HRT others**	0.0056 [-0.9757 - 0.9869]	-0.0000 [-0.0005 - 0.0004]	-0.0001 [-0.0036 - 0.0033]	-0.0003 [-0.0020 - 0.0013]	0.0052 [-0.8926 - 0.9029]	0.0115 [-0.7714 - 0.7943]
**Ever used HRT yes**	0.0001 [-0.0012 - 0.0014]	0.0002 [-0.0013 - 0.0017]	-0.0000 [-0.0012 - 0.0011]	0.0011 [-0.0020 - 0.0042]	0.0011 [-0.0027 - 0.0050]	0.0027 [-0.0064 - 0.0119]
**Had menopause hysterectomy**	0.0003 [-0.0019 - 0.0026]	-0.0018 [-0.0073 - 0.0037]	0.0008 [-0.0016 - 0.0033]	-0.0030 [-0.0100 - 0.0040]	-0.0016 [-0.0057 - 0.0025]	-0.0342 [-0.1142 - 0.0458]
**Had menopause no**	-0.0006 [-0.0043 - 0.0031]	0.0049 [-0.0050 - 0.0147]	-0.0041 [-0.0130 - 0.0047]	0.0011 [-0.0019 - 0.0041]	0.0010 [-0.0028 - 0.0047]	0.0046 [-0.0069 - 0.0162]
**Had menopause others**	0.0008 [-0.0033 - 0.0050]	-0.0026 [-0.0082 - 0.0029]	-0.0006 [-0.0033 - 0.0020]	-0.0003 [-0.0018 - 0.0012]	0.0011 [-0.0031 - 0.0053]	-0.0014 [-0.0049 - 0.0021]
**Had menopause yes**	-0.0000 [-0.0025 - 0.0024]	0.0014 [-0.0026 - 0.0054]	0.0001 [-0.0019 - 0.0020]	-0.0020 [-0.0061 - 0.0022]	-0.0014 [-0.0053 - 0.0024]	-0.0064 [-0.0225 - 0.0097]
**Oestradiol**	-0.0013 [-0.0118 - 0.0092]	0.0032 [-0.0094 - 0.0159]	0.0026 [-0.0137 - 0.0189]	-0.0001 [-0.0058 - 0.0055]	-0.0047 [-0.0199 - 0.0105]	0.0024 [-0.0092 - 0.0141]
**SHBG**	0.0116 [-0.0068 - 0.0301]	-0.0285 [-0.0608 - 0.0038]	-0.0015 [-0.0153 - 0.0122]	0.0103 [-0.0027 - 0.0232]	-0.0170 [-0.0403 - 0.0063]	-0.0543 [-0.1000 - -0.0087]

IEs and PMs with 95%CIs were calculated by mediation models to assess the role of hormones and menopause mediators in female participants. Abbreviations: CI, Confidence Interval; IE, Indirect Effect; PM, Proportion Mediated; SI, Social Isolation; HRT, Hormone Replacement Therapy; SHBG, Sex Hormone-Binding Globulin.

eTable13. Indirect Effect (95% Confidence Intervals) and Proportion Mediated (95% Confidence Intervals) of Individual Cancers Associated with Inflammatory Markers in Female Participants.

Mediator	Bladder Cancer	Breast Cancer	Lung Cancer	Ovarian Cancer	Stomach Cancer	Uterine Cancer
**Indirect Effect (95% Confidence Intervals)**						
**CRP**	0.0001 [-0.0013 - 0.0015]	-0.0000 [-0.0002 - 0.0002]	-0.0000 [-0.0013 - 0.0013]	0.0001 [-0.0013 - 0.0014]	-0.0000 [-0.0009 - 0.0009]	0.0000 [-0.0004 - 0.0005]
**Lym**	0.0006 [-0.0008 - 0.0019]	0.0004 [-0.0002 - 0.0010]	0.0006 [-0.0004 - 0.0015]	-0.0001 [-0.0011 - 0.0009]	0.0005 [-0.0008 - 0.0019]	-0.0000 [-0.0008 - 0.0008]
**Lym mono ratio**	0.0003 [-0.0013 - 0.0018]	-0.0000 [-0.0004 - 0.0003]	-0.0004 [-0.0014 - 0.0007]	-0.0005 [-0.0022 - 0.0013]	0.0007 [-0.0007 - 0.0021]	0.0002 [-0.0006 - 0.0009]
**Mono**	-0.0002 [-0.0009 - 0.0005]	-0.0001 [-0.0005 - 0.0003]	-0.0001 [-0.0008 - 0.0005]	-0.0001 [-0.0005 - 0.0003]	0.0001 [-0.0009 - 0.0011]	-0.0001 [-0.0006 - 0.0004]
**Neu**	0.0093 [-0.0072 - 0.0259]	0.0059 [0.0030 - 0.0088]	0.0156 [0.0096 - 0.0216]	0.0069 [-0.0022 - 0.0159]	0.0149 [-0.0018 - 0.0315]	0.0066 [-0.0006 - 0.0138]
**Neu lym ratio**	0.0021 [-0.0059 - 0.0102]	0.0014 [-0.0004 - 0.0032]	0.0041 [0.0015 - 0.0067]	0.0015 [-0.0036 - 0.0067]	0.0011 [-0.0097 - 0.0120]	0.0018 [-0.0022 - 0.0058]
**PLT**	0.0029 [-0.0075 - 0.0133]	-0.0009 [-0.0027 - 0.0008]	0.0043 [0.0004 - 0.0082]	-0.0016 [-0.0071 - 0.0039]	0.0133 [0.0032 - 0.0234]	-0.0006 [-0.0050 - 0.0038]
**PLT lym ratio**	-0.0007 [-0.0074 - 0.0059]	-0.0010 [-0.0024 - 0.0003]	-0.0004 [-0.0029 - 0.0020]	-0.0012 [-0.0049 - 0.0025]	0.0002 [-0.0022 - 0.0027]	-0.0009 [-0.0038 - 0.0021]
**Systemic inflammation**	0.0023 [-0.0091 - 0.0138]	0.0010 [-0.0014 - 0.0034]	0.0060 [0.0026 - 0.0094]	0.0011 [-0.0061 - 0.0082]	0.0047 [-0.0046 - 0.0139]	0.0016 [-0.0040 - 0.0072]
**WBC**	0.0103 [-0.0014 - 0.0219]	0.0053 [0.0029 - 0.0077]	0.0123 [0.0076 - 0.0169]	0.0039 [-0.0035 - 0.0113]	0.0107 [-0.0002 - 0.0216]	0.0041 [-0.0018 - 0.0099]
**Proportion Mediated (95% Confidence Intervals)**						
**CRP**	0.0001 [-0.0022 - 0.0023]	-0.0002 [-0.0023 - 0.0019]	-0.0001 [-0.0047 - 0.0044]	0.0001 [-0.0027 - 0.0029]	-0.0000 [-0.0013 - 0.0013]	0.0001 [-0.0012 - 0.0015]
**Lym**	0.0009 [-0.0014 - 0.0032]	0.0034 [-0.0031 - 0.0100]	0.0019 [-0.0017 - 0.0056]	-0.0002 [-0.0023 - 0.0019]	0.0008 [-0.0013 - 0.0029]	-0.0001 [-0.0026 - 0.0024]
**Lym mono ratio**	0.0004 [-0.0022 - 0.0030]	-0.0001 [-0.0034 - 0.0031]	-0.0012 [-0.0049 - 0.0024]	-0.0010 [-0.0047 - 0.0026]	0.0010 [-0.0012 - 0.0032]	0.0005 [-0.0019 - 0.0030]
**Mono**	-0.0003 [-0.0014 - 0.0009]	-0.0010 [-0.0050 - 0.0030]	-0.0004 [-0.0027 - 0.0019]	-0.0001 [-0.0010 - 0.0007]	0.0001 [-0.0013 - 0.0015]	-0.0003 [-0.0018 - 0.0012]
**Neu**	0.0150 [-0.0135 - 0.0435]	0.0552 [-0.0019 - 0.1122]	0.0544 [0.0177 - 0.0912]	0.0141 [-0.0060 - 0.0343]	0.0217 [-0.0066 - 0.0500]	0.0210 [-0.0064 - 0.0484]
**Neu lym ratio**	0.0034 [-0.0097 - 0.0166]	0.0129 [-0.0074 - 0.0332]	0.0143 [0.0022 - 0.0264]	0.0032 [-0.0075 - 0.0139]	0.0016 [-0.0142 - 0.0175]	0.0058 [-0.0076 - 0.0191]
**PLT**	0.0047 [-0.0124 - 0.0218]	-0.0087 [-0.0272 - 0.0097]	0.0150 [-0.0008 - 0.0308]	-0.0033 [-0.0148 - 0.0082]	0.0194 [-0.0003 - 0.0391]	-0.0020 [-0.0163 - 0.0122]
**PLT lym ratio**	-0.0011 [-0.0119 - 0.0096]	-0.0097 [-0.0252 - 0.0058]	-0.0016 [-0.0101 - 0.0070]	-0.0025 [-0.0101 - 0.0052]	0.0003 [-0.0032 - 0.0039]	-0.0027 [-0.0123 - 0.0068]
**Systemic inflammation**	0.0037 [-0.0149 - 0.0224]	0.0093 [-0.0145 - 0.0331]	0.0209 [0.0042 - 0.0376]	0.0022 [-0.0127 - 0.0170]	0.0068 [-0.0074 - 0.0210]	0.0051 [-0.0131 - 0.0233]
**WBC**	0.0165 [-0.0054 - 0.0383]	0.0492 [-0.0010 - 0.0994]	0.0428 [0.0140 - 0.0715]	0.0081 [-0.0077 - 0.0239]	0.0156 [-0.0035 - 0.0346]	0.0131 [-0.0078 - 0.0340]

IEs and PMs with 95%CIs were calculated by mediation models to assess the role of inflammatory markers mediators in female participants. Abbreviations: CI, Confidence Interval; IE, Indirect Effect; PM, Proportion Mediated; SI, Social Isolation; Lym, Lymphocytes; Mono, Monocytes; Neu, Neutrophils; PLT, Platelets; WBC, White Blood Cells; Lym Mono Ratio, Lymphocyte to Monocyte Ratio; Neu Lym Ratio, Neutrophil to Lymphocyte Ratio; PLT Lym Ratio, Platelet to Lymphocyte Ratio.

eTable14. Indirect Effect (95% Confidence Intervals) and Proportion Mediated (95% Confidence Intervals) of Individual Cancers Associated with Inflammatory Markers in Male Participants.

	Bladder cancer	
**Mediator**	**IE 95%CI**	**PM 95%CI**
**CRP**	0.0009 [-0.0016 - 0.0034]	0.0024 [-0.0049 - 0.0097]
**Lym**	-0.0015 [-0.0043 - 0.0013]	-0.0042 [-0.0129 - 0.0045]
**Lym mono ratio**	0.0001 [-0.0004 - 0.0006]	0.0003 [-0.0011 - 0.0017]
**Mono**	0.0000 [-0.0003 - 0.0003]	0.0000 [-0.0008 - 0.0008]
**Neu**	0.0089 [-0.0016 - 0.0193]	0.0250 [-0.0109 - 0.0608]
**Neu kym ratio**	0.0021 [-0.0044 - 0.0086]	0.0059 [-0.0130 - 0.0248]
**PLT**	-0.0014 [-0.0050 - 0.0022]	-0.0039 [-0.0144 - 0.0067]
**PLT lym ratio**	-0.0038 [-0.0121 - 0.0044]	-0.0108 [-0.0357 - 0.0141]
**Systemic inflammation**	0.0010 [-0.0069 - 0.0090]	0.0029 [-0.0196 - 0.0254]
**WBC**	0.0058 [-0.0001 - 0.0116]	0.0162 [-0.0051 - 0.0374]

IEs and PMs with 95%CIs were calculated by mediation models to assess the role of inflammatory markers mediators in male participants. Abbreviations: CI, Confidence Interval; IE, Indirect Effect; PM, Proportion Mediated; SI, Social Isolation; Lym, Lymphocytes; Mono, Monocytes; Neu, Neutrophils; PLT, Platelets; WBC, White Blood Cells; Lym Mono Ratio, Lymphocyte to Monocyte Ratio; Neu lym Ratio, Neutrophil to Lymphocyte Ratio; PLT Lym Ratio, Platelet to Lymphocyte Ratio.

eTable15. Baseline Characteristics of the Study Population Stratified by Exposures in Sensitive Analysis (Baseline in 2006.10).

Variable Label	Label	Overall, N = 421,537	No social isolation, N = 401,659	Social isolation, N = 19,878	P-Value	No loneliness, N = 396,164	Loneliness, N = 25,373	P-value
Age, years		56.00 ± 8.06	55.95 ± 8.08	56.82 ± 7.66	1.93e-55	56.03 ± 8.07	55.44 ± 7.88	1.32e-26
Sex, Male,n (%)		159,904 (46%)	151,354 (46%)	8,550 (43%)	1.37e-08	152,078 (45%)	7,826 (51%)	3.23e-53
Ethnicity,White,n (%)		335,993 (96%)	317,193 (96%)	18,800 (95%)	1.85e-10	321,441 (96%)	14,552 (94%)	1.52e-23
Assessment Center					2.71e-07			1.35e-02
	English,n (%)	322,814 (92%)	304,575 (92%)	18,239 (92%)		308,690 (92%)	14,124 (92%)	
	Scotland,n (%)	12,832 (4%)	12,049 (4%)	783 (4%)		12,236 (4%)	596 (4%)	
	Wales,n (%)	15,018 (4%)	14,295 (4%)	723 (4%)		14,336 (4%)	682 (4%)	
Employment					7.49e-325			2.32e-415
	Employed,n (%)	214,326 (61%)	203,116 (61%)	11,210 (57%)		205,470 (61%)	8,856 (57%)	
	Others,n (%)	27,647 (8%)	25,055 (8%)	2,592 (13%)		25,272 (8%)	2,375 (15%)	
	Retired,n (%)	108,691 (31%)	102,748 (31%)	5,943 (30%)		104,520 (31%)	4,171 (27%)	
College/university degree, n (%)		177,702 (51%)	168,947 (51%)	8,755 (44%)	5.28e-126	171,044 (51%)	6,658 (43%)	7.90e-114
Income					2.78e-2806			4.37e-604
	High,n (%)	88,467 (25%)	86,897 (26%)	1,570 (8%)		86,137 (26%)	2,330 (15%)	
	Low,n (%)	61,479 (18%)	53,114 (16%)	8,365 (42%)		56,931 (17%)	4,548 (30%)	
	Medium,n (%)	161,760 (46%)	153,854 (46%)	7,906 (40%)		155,048 (46%)	6,712 (44%)	
	Unreported,n (%)	38,958 (11%)	37,054 (11%)	1,904 (10%)		37,146 (11%)	1,812 (12%)	
Smoking status					7.24e-650			3.19e-263
	Current,n (%)	34,205 (10%)	30,445 (9%)	3,760 (19%)		31,700 (9%)	2,505 (16%)	
	Never,n (%)	195,872 (56%)	186,386 (56%)	9,486 (48%)		188,207 (56%)	7,665 (50%)	
	Previous,n (%)	120,587 (34%)	114,088 (34%)	6,499 (33%)		115,355 (34%)	5,232 (34%)	
Alcohol status					6.23e-437			1.73e-100
	Current,n (%)	326,674 (93%)	309,533 (94%)	17,141 (87%)		312,832 (93%)	13,842 (90%)	
	Never,n (%)	12,854 (4%)	11,714 (4%)	1,140 (6%)		12,120 (4%)	734 (5%)	
	Previous,n (%)	11,136 (3%)	9,672 (3%)	1,464 (7%)		10,310 (3%)	826 (5%)	
Family cancer history, n (%)		109,647 (31%)	103,312 (31%)	6,335 (32%)	3.62e-02	104,749 (31%)	4,898 (32%)	1.53e-01
Townsend Deprivation Score		-1.49 ± 2.96	-1.59 ± 2.90	0.17 ± 3.42	3.25e-1353	-1.53 ± 2.94	-0.63 ± 3.35	8.05e-334
Sun exposure time, hours/day		2.82 ± 1.89	2.82 ± 1.88	2.77 ± 2.03	2.04e-03	2.82 ± 1.88	2.84 ± 2.08	4.22e-02
Diet Score		2.18 ± 1.21	2.18 ± 1.21	2.23 ± 1.21	3.29e-04	2.19 ± 1.21	2.02 ± 1.20	3.22e-88
Sleep Score		2.39 ± 0.74	2.41 ± 0.74	2.21 ± 0.81	2.58e-282	2.41 ± 0.73	2.06 ± 0.84	1.27e-635
Depress mood					1.09e-523			2.63e-2740
	High,n (%)	15,141 (4%)	13,170 (4%)	1,971 (10%)		12,070 (4%)	3,071 (20%)	
	Low,n (%)	324,790 (93%)	307,720 (93%)	17,070 (86%)		313,271 (93%)	11,519 (75%)	
	Unreported,n (%)	10,733 (3%)	10,029 (3%)	704 (4%)		9,921 (3%)	812 (5%)	
BMI (kg/m2)		27.21 ± 4.78	27.18 ± 4.73	27.76 ± 5.55	6.79e-54	27.17 ± 4.74	28.18 ± 5.52	2.13e-121
MAP(mmHg)		101.14 ± 12.45	101.12 ± 12.44	101.48 ± 12.68	4.76e-05	101.16 ± 12.45	100.81 ± 12.41	1.68e-04
Grip strength, kg		31.07 ± 11.02	31.18 ± 11.02	29.26 ± 10.81	7.62e-167	31.09 ± 11.00	30.71 ± 11.39	9.70e-09
Overall Health Rating		2.92 ± 0.72	2.93 ± 0.71	2.66 ± 0.82	1.66e-640	2.94 ± 0.71	2.51 ± 0.83	6.44e-1045

Baseline variates were presented as means ± standard error or median (interquartile range) for continuous variables and frequency (percentages) for categorical variables. Continuous variables were assessed for statistical differences using two-sample T-tests, ANOVA tests, or Mann–Whitney U tests. Categorical variables were evaluated for differences using the $\chi 2$ test. Abbreviations: ANOVA, Analysis of Variance; BMI, Body Mass Index; MAP, Mean Arterial Pressure; χ^2, Chi-Squared; SI, Social Isolation.

eTable16: Overall Comparison of Included vs Excluded Participants

Variable	Level	Excluded (n=147828)	Included (n=354537)	P-value	Test	SMD
**Isolation**		0.08 (0.27)	0.06 (0.23)	< 1.00e-300		0.085
**Loneliness**		0.06 (0.24)	0.04 (0.21)	< 1.00e-300		0.064
**Indicator**		0.35 (0.56)	0.18 (0.47)	< 1.00e-300		0.326
**Age event**		63.88 [55.80, 71.67]	67.75 [60.28, 73.59]	< 1.00e-300	nonnorm	0.371
**Age entry**		59.00 [51.00, 64.00]	57.00 [50.00, 63.00]	2.40e-173	nonnorm	0.081
**Sex**	**Female**	82722 (56.0)	190576 (53.8)	< 1.00e-300		0.044
	**Male**	65106 (44.0)	163961 (46.2)			
**Race**	**Non white**	12011 (8.3)	14177 (4.0)	< 1.00e-300		0.179
	**White**	133048 (91.7)	340360 (96.0)			
**BMI**		26.92 [24.17, 30.22]	26.63 [24.06, 29.75]	5.91e-72	nonnorm	0.009
**UK Center**	**English**	116008 (80.5)	325924 (91.9)	< 1.00e-300		0.420
	**Scotland**	22845 (15.9)	12992 (3.7)			
	**Wales**	5183 (3.6)	15621 (4.4)			
**Education**	**College/university degree**	58727 (41.3)	178566 (50.4)	< 1.00e-300		0.184
	**Not college degree**	83611 (58.7)	175971 (49.6)			
**Employment**	**Employed**	75650 (51.9)	211397 (59.6)	< 1.00e-300		0.179
	**Others**	17855 (12.3)	28422 (8.0)			
	**Retired**	52245 (35.8)	114718 (32.4)			
**Smoking status**	**Current**	17622 (12.2)	35339 (10.0)	< 1.00e-300		0.070
	**Never**	78195 (54.0)	195266 (55.1)			
	**Previous**	49084 (33.9)	123932 (35.0)			
**Alcohol status**	**Current**	130463 (89.3)	329778 (93.0)	< 1.00e-300		0.139
	**Never**	9353 (6.4)	13025 (3.7)			
	**Previous**	6359 (4.4)	11734 (3.3)			
**Depress mood**	**High**	8740 (5.9)	15551 (4.4)	< 1.00e-300		0.265
	**Low**	125798 (85.1)	328107 (92.5)			
	**Unreported**	13290 (9.0)	10879 (3.1)			
**Townsend Deprivation index**		-1.72 [-3.44, 1.36]	-2.27 [-3.70, 0.20]	< 1.00e-300	nonnorm	0.202
**Family cancer**	**Yes**	96901 (69.7)	243454 (68.7)	< 1.00e-300		0.022
	**No**	42187 (30.3)	111083 (31.3)			
**Overall Health rating**		3.00 [2.00, 3.00]	3.00 [3.00, 3.00]	< 1.00e-300	nonnorm	0.250
**Hand grip**		28.00 [21.50, 37.00]	29.50 [23.00, 39.00]	< 1.00e-300	nonnorm	0.123
**Day exposure**		2.50 [1.50, 4.00]	2.50 [1.50, 3.50]	< 1.00e-300	nonnorm	0.107
**Diet score**		2.00 [1.00, 3.00]	2.00 [1.00, 3.00]	< 1.00e-300	nonnorm	0.018
**Sleep score**		3.00 [2.00, 3.00]	3.00 [2.00, 3.00]	< 1.00e-300	nonnorm	0.080
**MAP**		101.33 [93.00, 110.00]	100.67 [92.33, 109.33]	< 1.00e-300	nonnorm	0.052
**Income**	**High**	21618 (14.6)	87548 (24.7)	< 1.00e-300		0.441
	**Low**	32687 (22.1)	64489 (18.2)			
	**Medium**	55992 (37.9)	162895 (45.9)			
	**Unreported**	37531 (25.4)	39605 (11.2)			

Categorical variables are presented as n (%); continuous variables are presented as mean (SD) for normally distributed data or median [IQR] for non-normally distributed data. P-values were calculated using chi-square tests for categorical variables and Wilcoxon rank-sum tests for non-normal continuous variables. SMD were computed to assess balance, with SMD <0.3 indicating negligible difference, >0.3 indicating moderate to large difference. Variables with an SMD exceeding 0.3 are highlighted in red to indicate notable imbalance. Abbreviations: SD, Standard Deviation; IQR, Interquartile Range; SMD, Standardized Mean Differences; BMI, Body Mass Index; MAP, Mean Arterial Pressure

eTable17: Comparison of Included vs Subgroup Excluded Due to Missing Covariates or Exposures

Variable	Level	Included (n=354537)	Missing Subgroup (n=114488)	P-value	Test	SMD
**n**		354537	114488			
**Indicator**		0.18 (0.47)	0.26 (0.54)	< 1.00e-300		0.156
**Age event**		67.75 [60.28, 73.59]	66.98 [59.00, 73.41]	< 1.00e-300	nonnorm	0.099
**Income**	**High**	87548 (24.7)	15296 (13.4)	< 1.00e-300		0.523
	**Low**	64489 (18.2)	25275 (22.1)			
	**Medium**	162895 (45.9)	40962 (35.8)			
	**Unreported**	39605 (11.2)	32955 (28.8)			
**Townsend deprivation index**		-1.48 (2.97)	-0.70 (3.39)	< 1.00e-300		0.246
**Depress mood**	**High**	15551 (4.4)	7280 (6.4)	< 1.00e-300		0.322
	**Low**	328107 (92.5)	95057 (83.0)			
	**Unreported**	10879 (3.1)	12151 (10.6)			
**Isolation**		0.06 (0.23)	0.08 (0.28)	< 1.00e-300		0.097
**Loneliness**		0.04 (0.21)	0.07 (0.25)	< 1.00e-300		0.089

Categorical variables are presented as n (%); continuous variables are presented as mean (SD) for normally distributed data or median [IQR] for non-normally distributed data. P-values were calculated using chi-square tests for categorical variables and Wilcoxon rank-sum tests for non-normal continuous variables. SMD were computed to assess balance, with SMD <0.3 indicating negligible difference, >0.3 indicating moderate to large difference. Variables with an SMD exceeding 0.3 are highlighted in red to indicate notable imbalance. Abbreviations: SMD, Standardized Mean Differences; SD, Standard Deviation; IQR, Interquartile Range; SMD, Standardized Mean Differences.

eTable18: Logistic Regression for Missing Data (NA_any)

Term	OR	95% CI Lower	95% CI Upper
**Income High**	1	Ref	
**Income Low**	2.3120736	2.2560357	2.3695703
**Income Medium**	1.4795781	1.4481012	1.5118051
**Income Unreported**	4.4758375	4.3662571	4.5883309
**Depress mood Low**	1	Ref	
**Depress mood High**	1.4789435	1.4344879	1.5246276
**Depress mood Unreported**	3.5413343	3.4440784	3.6413140
**UK Center England**	1	Ref	
**UK Center Scotland**	7.6536092	7.4768568	7.8347760
**UK Center Wales**	0.9890250	0.9533299	1.0258203

Logistic regression analysis of factors associated with missingness of any covariate or exposure in the UK Biobank cohort (n=502,365). The model examines the association between income levels, depressive mood status, UK Biobank assessment centers, and the probability of having missing data. Results are presented as OR with 95%CI. Abbreviations: OR, Odds Ratio; CI, Confidence Interval; SES, Socioeconomic Status.

eTable19. Stability of Social Isolation and Loneliness Across Instances Subset with Repeated Assessments.

Items		Social Isolation		Loneliness	
**Pair**	**Pattern**	**N**	**%**	**N**	**%**
**0-1**	**Never**	18,130	92.1	17,740	94.2
	**Incident**	620	3.2	391	2.1
	**Remitted**	440	2.2	426	2.3
	**Persistent**	493	2.5	281	1.5
	**Overall**	19,683	100	18,838	100
**Agreement %**		94.6		95.7	
**0-2**	**Never**	58,163	91.7	56,604	94.1
	**Incident**	2,498	3.9	1,454	2.4
	**Remitted**	1,620	2.6	1,391	2.3
	**Persistent**	1,144	1.8	713	1.2
	**Overall**	63,425	100	60,162	100
**Agreement %**		93.5		95.3	
**0-3**	**Never**	4,733	90.7	4,705	94.1
	**Incident**	276	5.3	132	2.6
	**Remitted**	113	2.2	104	2.1
	**Persistent**	95	1.8	57	1.1
	**Overall**	5,217	100	4,998	100
**Agreement %**		92.5		95.3	

Pairs 0-1, 0-2, and 0-3 are instance 1,2,3 compared to baseline. Patterns are defined as: Never: Non-exposed at both instances (0-0). Incident: Non-exposed at first instance, exposed at second (0-1). Remitted: Exposed at first instance, non-exposed at second (1-0). Persistent: Exposed at both instances (1-1). N and % are counts and percentages per pattern, with Overall as complete-case sample size per pair. Agreement % = [(Never N + Persistent N) / Overall N] * 100, reflecting the proportion of participants with consistent classification. Analyses used complete cases due to high missingness. Pair 0-3 results may be less reliable due to small sample size (N<6,000). Abbreviations: N, Number; SI, Social Isolation.

eTable20. Proportion and Trend of Social Isolation and Loneliness by Instance.

	Social Isolation		Loneliness	
**Instance**	**N**	**Proportion(%)**	**N**	**Proportion (%)**
**0**	491,090	6.4	476,108	4.9
**1**	20,243	5.7	19,456	3.6
**2**	64,573	5.8	61,897	3.6
**3**	5,316	7.1	5,139	3.8
	**Note: Linear trend slope = 0.22% per instance, p = 0.56, no significant change over time.**		**Note: Linear trend slope = -0.32% per instance, p = 0.31, indicating no significant change over time.**	

Proportion (%) = percentage of participants with exposure per instance. Linear trend slope and p-value from regression of proportions on instance number; $p > 0.05$ indicates no significant change over time. Instance 3 results may be less reliable due to small sample size.

Supplementary Figures

eFigure1. Associations of SI., Loneliness with Organ-Specific Cancer Risk by Cancer Historical Types in Females (Model3).

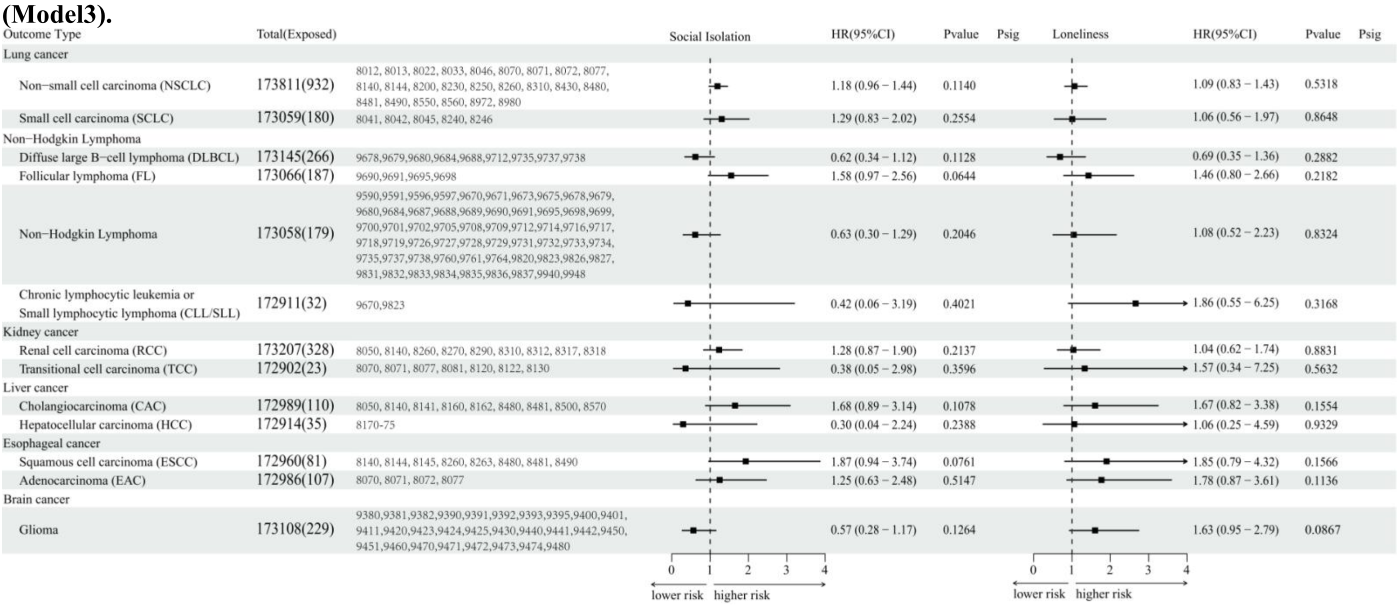

Outcome Type	Total(Exposed)		Social Isolation HR(95%CI)	Pvalue	Psig	Loneliness HR(95%CI)	Pvalue	Psig
Lung cancer								
Non−small cell carcinoma (NSCLC)	173811(932)	8012, 8013, 8022, 8033, 8046, 8070, 8071, 8072, 8077, 8140, 8144, 8200, 8230, 8250, 8260, 8310, 8430, 8480, 8481, 8490, 8550, 8560, 8972, 8980	1.18 (0.96 − 1.44)	0.1140		1.09 (0.83 − 1.43)	0.5318	
Small cell carcinoma (SCLC)	173059(180)	8041, 8042, 8045, 8240, 8246	1.29 (0.83 − 2.02)	0.2554		1.06 (0.56 − 1.97)	0.8648	
Non−Hodgkin Lymphoma								
Diffuse large B−cell lymphoma (DLBCL)	173145(266)	9678,9679,9680,9684,9688,9712,9735,9737,9738	0.62 (0.34 − 1.12)	0.1128		0.69 (0.35 − 1.36)	0.2882	
Follicular lymphoma (FL)	173066(187)	9690,9691,9695,9698	1.58 (0.97 − 2.56)	0.0644		1.46 (0.80 − 2.66)	0.2182	
Non−Hodgkin Lymphoma	173058(179)	9590,9591,9596,9597,9670,9671,9673,9675,9678,9679, 9680,9684,9687,9688,9689,9690,9691,9695,9698,9699, 9700,9701,9702,9705,9708,9709,9712,9714,9716,9717, 9718,9719,9726,9727,9728,9729,9731,9732,9733,9734, 9735,9737,9738,9760,9761,9764,9820,9823,9826,9827, 9831,9832,9833,9834,9835,9836,9837,9940,9948	0.63 (0.30 − 1.29)	0.2046		1.08 (0.52 − 2.23)	0.8324	
Chronic lymphocytic leukemia or Small lymphocytic lymphoma (CLL/SLL)	172911(32)	9670,9823	0.42 (0.06 − 3.19)	0.4021		1.86 (0.55 − 6.25)	0.3168	
Kidney cancer								
Renal cell carcinoma (RCC)	173207(328)	8050, 8140, 8260, 8270, 8290, 8310, 8312, 8317, 8318	1.28 (0.87 − 1.90)	0.2137		1.04 (0.62 − 1.74)	0.8831	
Transitional cell carcinoma (TCC)	172902(23)	8070, 8071, 8077, 8081, 8120, 8122, 8130	0.38 (0.05 − 2.98)	0.3596		1.57 (0.34 − 7.25)	0.5632	
Liver cancer								
Cholangiocarcinoma (CAC)	172989(110)	8050, 8140, 8141, 8160, 8162, 8480, 8481, 8500, 8570	1.68 (0.89 − 3.14)	0.1078		1.67 (0.82 − 3.38)	0.1554	
Hepatocellular carcinoma (HCC)	172914(35)	8170-75	0.30 (0.04 − 2.24)	0.2388		1.06 (0.25 − 4.59)	0.9329	
Esophageal cancer								
Squamous cell carcinoma (ESCC)	172960(81)	8140, 8144, 8145, 8260, 8263, 8480, 8481, 8490	1.87 (0.94 − 3.74)	0.0761		1.85 (0.79 − 4.32)	0.1566	
Adenocarcinoma (EAC)	172986(107)	8070, 8071, 8072, 8077	1.25 (0.63 − 2.48)	0.5147		1.78 (0.87 − 3.61)	0.1136	
Brain cancer								
Glioma	173108(229)	9380,9381,9382,9390,9391,9392,9393,9395,9400,9401, 9411,9420,9423,9424,9425,9430,9440,9441,9442,9450, 9451,9460,9470,9471,9472,9473,9474,9480	0.57 (0.28 − 1.17)	0.1264		1.63 (0.95 − 2.79)	0.0867	

Forest plot illustrates the CSHR of SI and loneliness on specific cancer types in females. CSHR and 95%CI were estimated by cause-specific Cox proportional hazards model using age as the underlying time variable. The model was adjusted for age, sex, race/ethnicity, assessment center, employment, college/university degree, sun exposure time, , Townsend deprivation score, smoking status, alcohol use, BMI, grip strength, family history of cancer, MAP, overall health rating, healthy diet score, healthy sleep score, and depressive mood. Psig was determined based on P value, with * for P < 0.05, ** for P < 0.01, *** for P < 0.001. Abbreviation: CSHR, Cause-Specific Hazard Ratio; sHR, Subdistribution Hazard Ratio; HR, Hazard Ratio; CI, Confidence Interval; SI, Social Isolation; BMI, Body Mass Index; MAP, Mean Arterial Pressure.

eFigure2. Associations of SI, Loneliness with Organ-Specific Cancer Risk by Cancer Historical Types in Males (Model3).

Outcome Type	Total(Exposed)		Social Isolation	HR(95%CI)	Pvalue	Psig	Loneliness	HR(95%CI)	Pvalue	Psig
Lung cancer										
Non−small cell carcinoma (NSCLC)	173811(932)	8012, 8013, 8022, 8033, 8046, 8070, 8071, 8072, 8077, 8140, 8144, 8200, 8230, 8250, 8260, 8310, 8430, 8480, 8481, 8490, 8550, 8560, 8972, 8980		1.18 (0.96 − 1.44)	0.1140			1.09 (0.83 − 1.43)	0.5318	
Small cell carcinoma (SCLC)	173059(180)	8041, 8042, 8045, 8240, 8246		1.29 (0.83 − 2.02)	0.2554			1.06 (0.56 − 1.97)	0.8648	
Non−Hodgkin Lymphoma										
Diffuse large B−cell lymphoma (DLBCL)	173145(266)	9678,9679,9680,9684,9688,9712,9735,9737,9738		0.62 (0.34 − 1.12)	0.1128			0.69 (0.35 − 1.36)	0.2882	
Follicular lymphoma (FL)	173066(187)	9690,9691,9695,9698		1.58 (0.97 − 2.56)	0.0644			1.46 (0.80 − 2.66)	0.2182	
Non−Hodgkin Lymphoma	173058(179)	9590,9591,9596,9597,9670,9671,9673,9675,9678,9679, 9680,9684,9687,9688,9689,9690,9691,9695,9698,9699, 9700,9701,9702,9705,9708,9709,9712,9714,9716,9717, 9718,9719,9726,9727,9728,9729,9731,9732,9733,9734, 9735,9737,9738,9760,9761,9764,9820,9823,9826,9827, 9831,9832,9833,9834,9835,9836,9837,9940,9948		0.63 (0.30 − 1.29)	0.2046			1.08 (0.52 − 2.23)	0.8324	
Chronic lymphocytic leukemia or Small lymphocytic lymphoma (CLL/SLL)	172911(32)	9670,9823		0.42 (0.06 − 3.19)	0.4021			1.86 (0.55 − 6.25)	0.3168	
Kidney cancer										
Renal cell carcinoma (RCC)	173207(328)	8050, 8140, 8260, 8270, 8290, 8310, 8312, 8317, 8318		1.28 (0.87 − 1.90)	0.2137			1.04 (0.62 − 1.74)	0.8831	
Transitional cell carcinoma (TCC)	172902(23)	8070, 8071, 8077, 8081, 8120, 8122, 8130		0.38 (0.05 − 2.98)	0.3596			1.57 (0.34 − 7.25)	0.5632	
Liver cancer										
Cholangiocarcinoma (CAC)	172989(110)	8050, 8140, 8141, 8160, 8162, 8480, 8481, 8500, 8570		1.68 (0.89 − 3.14)	0.1078			1.67 (0.82 − 3.38)	0.1554	
Hepatocellular carcinoma (HCC)	172914(35)	8170-75		0.30 (0.04 − 2.24)	0.2388			1.06 (0.25 − 4.59)	0.9329	
Esophageal cancer										
Squamous cell carcinoma (ESCC)	172960(81)	8140, 8144, 8145, 8260, 8263, 8480, 8481, 8490		1.87 (0.94 − 3.74)	0.0761			1.85 (0.79 − 4.32)	0.1566	
Adenocarcinoma (EAC)	172986(107)	8070, 8071, 8072, 8077		1.25 (0.63 − 2.48)	0.5147			1.78 (0.87 − 3.61)	0.1136	
Brain cancer										
Glioma	173108(229)	9380,9381,9382,9390,9391,9392,9393,9395,9400,9401, 9411,9420,9423,9424,9425,9430,9440,9441,9442,9450, 9451,9460,9470,9471,9472,9473,9474,9480		0.57 (0.28 − 1.17)	0.1264			1.63 (0.95 − 2.79)	0.0867	

0 1 2 3 4 — lower risk / higher risk

Forest plot illustrates the CSHR of SI and loneliness on specific cancer types in males. CSHR and 95%CI were estimated by cause-specific Cox proportional hazards model using age as the underlying time variable. The model was adjusted for age, sex, race/ethnicity, assessment center, employment, college/university degree, sun exposure time, Townsend deprivation score, smoking status, alcohol use, BMI, grip strength, family history of cancer, MAP, overall health rating, healthy diet score, healthy sleep score, and depressive mood. Psig was determined based on P value, with * for P < 0.05, ** for P < 0.01, *** for P < 0.001. Abbreviation: CSHR, Cause-Specific Hazard Ratio; sHR, Subdistribution Hazard Ratio; HR, Hazard Ratio; CI, Confidence Interval; SI, Social Isolation; BMI, Body Mass Index; MAP, Mean Arterial Pressure.

eFigure3. Log-Log Plot of SI and Loneliness.

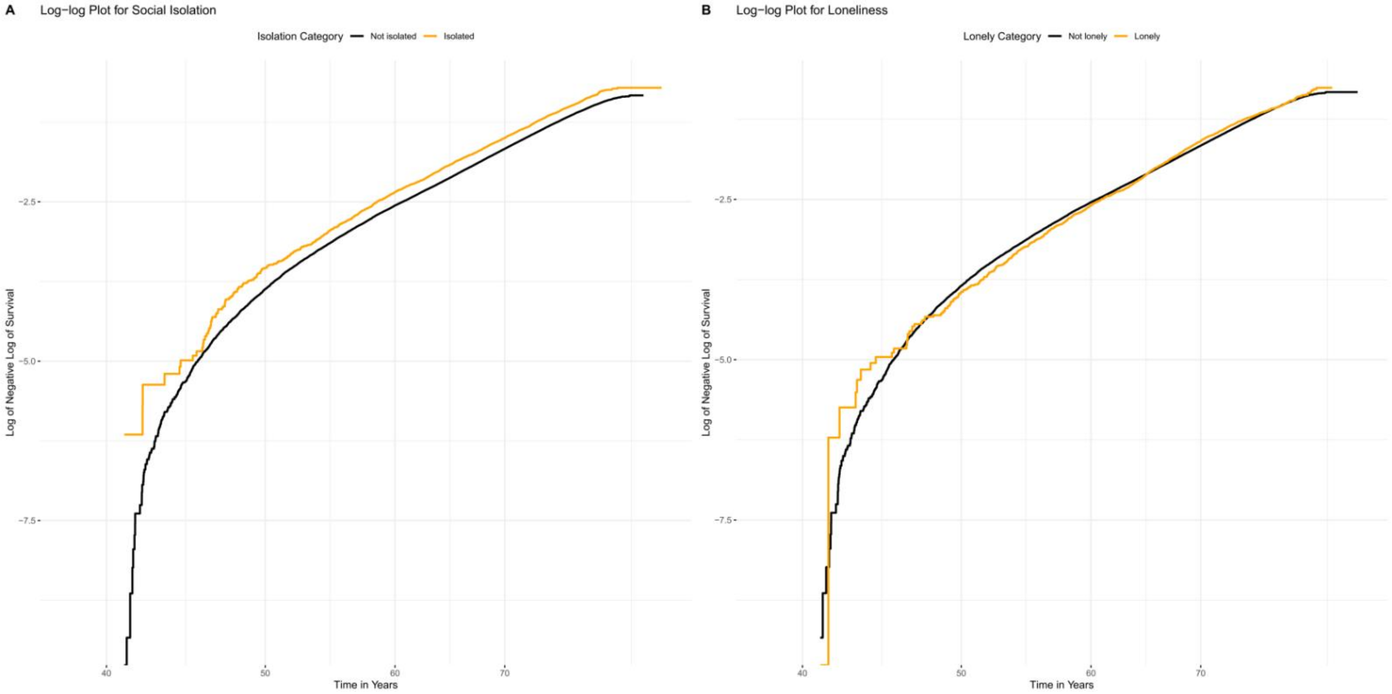

Panel (A) log-log plot for SI, (B) log-log plot for loneliness. Abbreviations: SI, Social Isolation.

eFigure4. Cumulative Incidence of Cancer by Social Isolation and Loneliness, Accounting for Competing Risks of Death, Over Up to 14 Years of Follow-up.

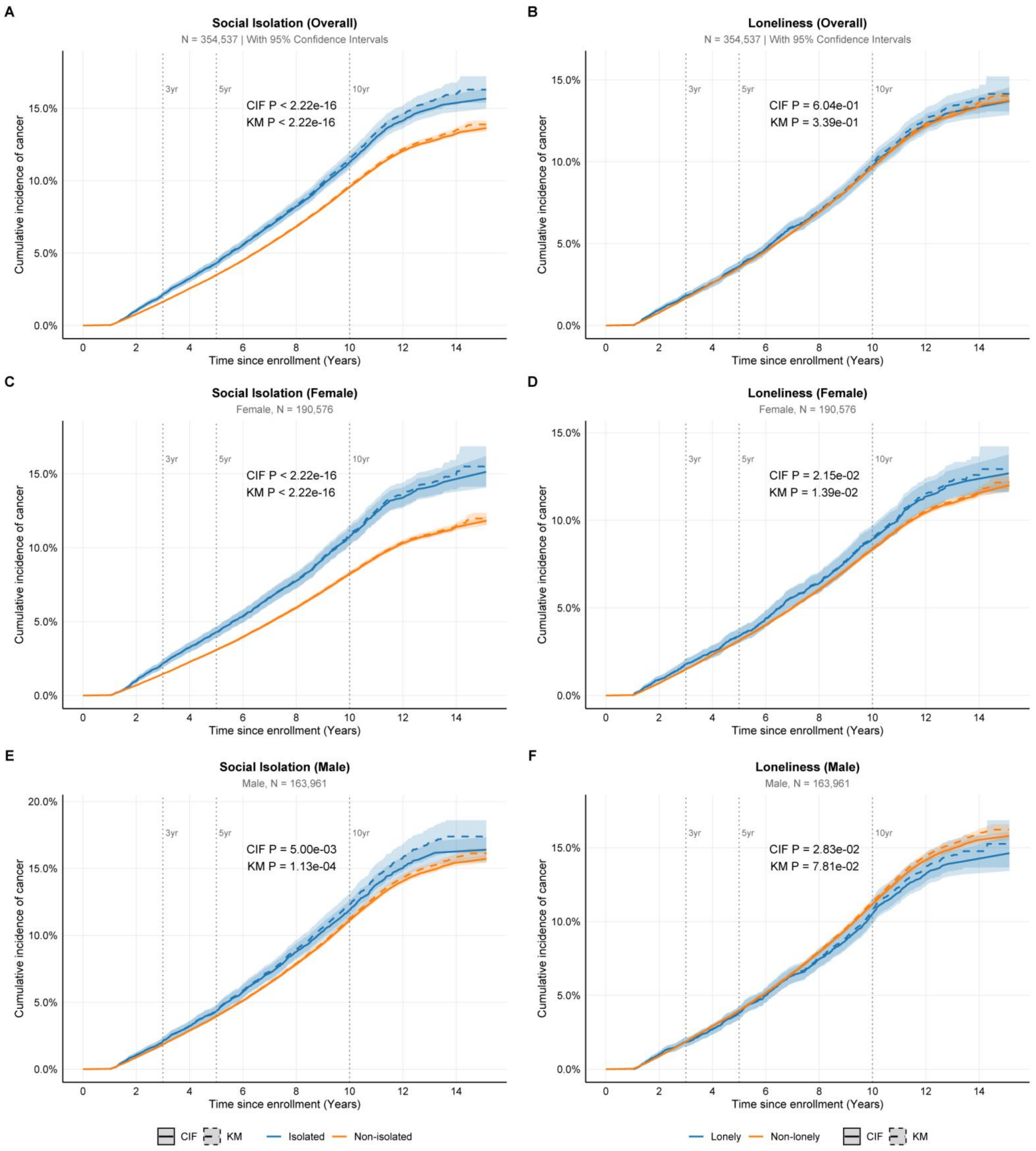

CIF curve (solid lines), and KM curve (dashed lines) illustrating estimates of association between SI or loneliness and cancer incidence in the overall population and stratified by sex. Panel (A) illustrates the effect of SI on survival in the overall population, (B) shows the effect of loneliness on survival in the overall population, (C) shows the effect of SI on survival in males, (D) shows the effect of loneliness on survival in males, (E) shows the effect of SI on survival in females, and (F) shows the effect of loneliness on survival in females. Shaded areas represent 95% confidence intervals. P-values (CIF and KM) for the difference between groups at 3, 5, and 10 years are annotated. Note: The KM method overestimates cancer incidence compared to the CIF method due to competing risks of death. Abbreviations: KM, Kaplan-Meier; CIF, Cumulative Incidence Functions; SI, Social Isolation.

eFigure5. Percentage of Excess Risk Mediated by Covariates for the Association of Loneliness or Social Isolation with Cancer Incidence, in the Overall Population and Stratified by Sex.

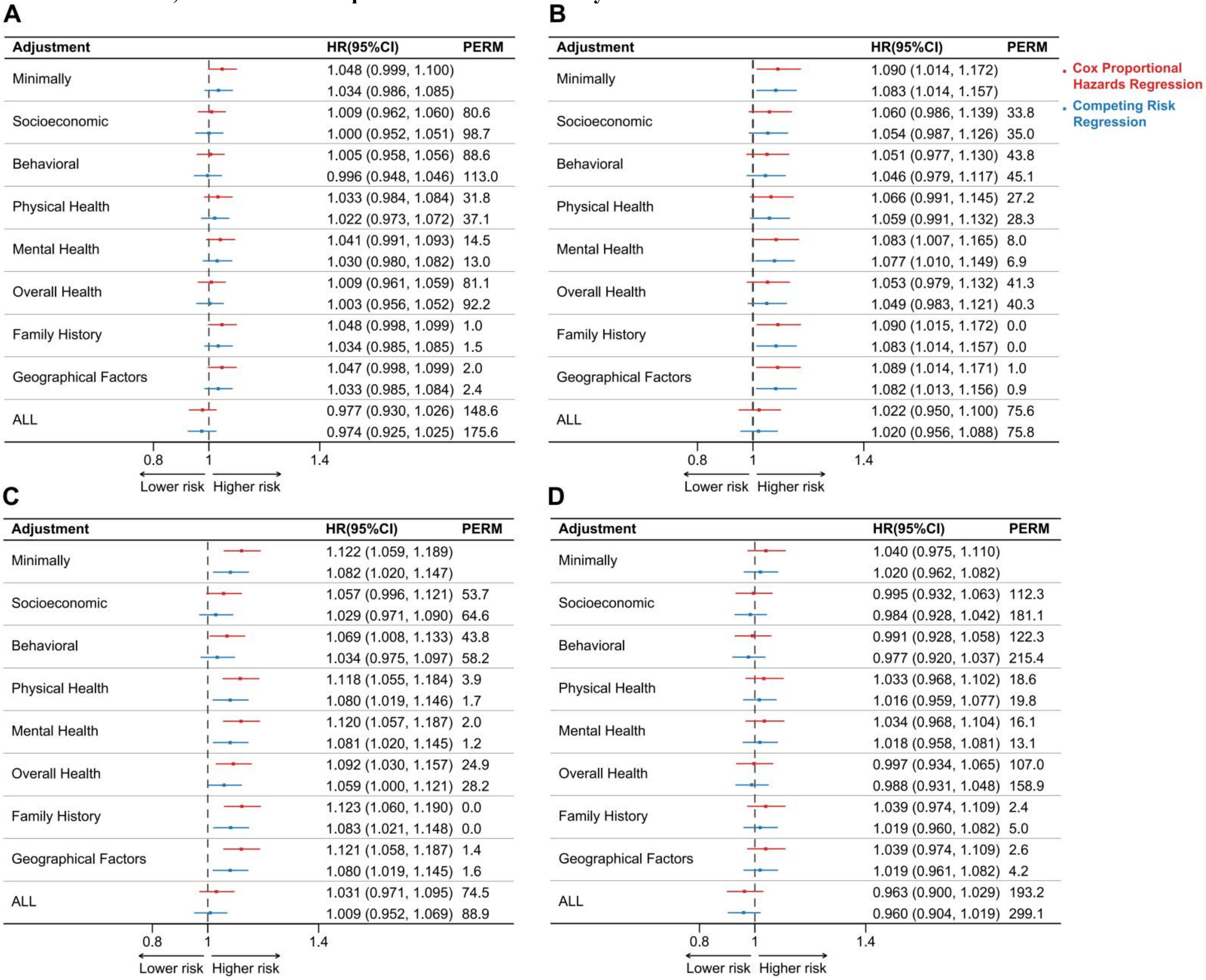

Panels (A) and (B) show the PERM by covariates for loneliness in the overall population (A) and females (B). Panels (C) and (D) show the PERM for SI (C) and loneliness (D) in males. The minimally adjusted model (adjusted for age, sex, and race/ethnicity) serves as the reference. HR estimates from the cause-specific Cox model (CSHR; red) and the competing risk model (sHR; blue) are shown. Abbreviations: CSHR, Cause-Specific Hazard Ratio; sHR, Subdistribution Hazard Ratio; PERM, Percentage of Excess Risk Attributable to Covariates; HR, Hazard Ratio; CI, Confidence Interval; SI, Social Isolation.

eFigure6. Associations of SI, Loneliness with Organ-Specific Cancer Risks in Female Participants (Model 1 and Model 2).

A

OutcomeType	Total(Exposed)	Model1	HR(95%CI)	Pvalue	Psig	Model2	HR(95%CI)	Pvalue	Psig
Melanoma	173979(1100)		0.67 (0.49 − 0.90)	8.872730e−03	**		0.77 (0.56 − 1.04)	0.0895533842	
Breast cancer	179627(6748)		1.15 (1.04 − 1.26)	4.879532e−03	**		1.14 (1.03 − 1.26)	0.0091456277	**
Lung cancer	174206(1327)		2.44 (2.09 − 2.85)	6.402490e−30	***		1.32 (1.12 − 1.55)	0.0008657266	***
Female genitalia cancer	173093(214)		1.23 (0.74 − 2.04)	4.336526e−01			1.20 (0.71 − 2.03)	0.5003029230	
Uterine cancer	173969(1090)		1.41 (1.14 − 1.75)	1.631876e−03	**		1.43 (1.15 − 1.79)	0.0013410084	**
Soft tissue cancer	173042(163)		1.55 (0.91 − 2.64)	1.063912e−01			1.45 (0.84 − 2.52)	0.1865595399	
Colorectal cancer	174719(1840)		1.14 (0.96 − 1.37)	1.417104e−01			1.08 (0.90 − 1.30)	0.4171650007	
Pancreatic cancer	173317(438)		1.56 (1.14 − 2.15)	6.074487e−03	**		1.35 (0.97 − 1.88)	0.0743707697	
Mouth cancer	172937(58)		2.45 (1.16 − 5.17)	1.874439e−02	*		1.42 (0.64 − 3.13)	0.3839115734	
Ovarian cancer	173576(697)		1.54 (1.19 − 1.99)	1.180130e−03	**		1.52 (1.16 − 1.99)	0.0021241815	**
Stomach cancer	173042(163)		2.43 (1.56 − 3.77)	8.362092e−05	***		1.89 (1.19 − 3.01)	0.0069476233	**
Esophageal cancer	173077(198)		1.76 (1.12 − 2.76)	1.465541e−02	*		1.53 (0.95 − 2.45)	0.0793462993	
Bladder cancer	173062(183)		2.32 (1.52 − 3.54)	9.379963e−05	***		1.70 (1.09 − 2.65)	0.0183838234	*
Kidney cancer	173261(382)		1.48 (1.04 − 2.10)	2.985129e−02	*		1.30 (0.90 − 1.87)	0.1651719921	
Eye cancer	172915(36)		1.93 (0.68 − 5.45)	2.172299e−01			1.42 (0.48 − 4.21)	0.5271749539	
Multiple myeloma	173171(292)		1.41 (0.93 − 2.12)	1.027275e−01			1.36 (0.89 − 2.08)	0.1591612481	
Leukemia	173253(374)		0.80 (0.51 − 1.28)	3.568575e−01			0.76 (0.48 − 1.22)	0.2570991390	
Tonsil cancer	172927(48)		1.09 (0.34 − 3.50)	8.905226e−01			0.70 (0.21 − 2.35)	0.5693422258	
Tongue cancer	172957(78)		1.31 (0.57 − 3.01)	5.275932e−01			0.87 (0.37 − 2.06)	0.7545703215	
Thyroid cancer	173102(223)		1.01 (0.58 − 1.77)	9.763909e−01			0.88 (0.50 − 1.56)	0.6597806205	
Brain cancer	173121(242)		0.53 (0.26 − 1.07)	7.435443e−02			0.54 (0.27 − 1.11)	0.0951573753	
Liver cancer	173035(156)		1.35 (0.77 − 2.38)	2.996817e−01			1.17 (0.65 − 2.11)	0.6052757166	
Lymphoma	173687(808)		0.94 (0.70 − 1.26)	6.589271e−01			0.85 (0.63 − 1.15)	0.3022784684	
Mesothelioma	172930(51)		0.91 (0.28 − 2.92)	8.727258e−01			0.63 (0.19 − 2.07)	0.4438348150	
Cervix Cancer	172970(91)		1.59 (0.77 − 3.29)	2.096644e−01			1.26 (0.60 − 2.67)	0.5420320847	
Biliary duct cancer	172995(116)		1.53 (0.82 − 2.85)	1.805371e−01			1.30 (0.68 − 2.46)	0.4291035994	
Anus Cancer	172983(104)		1.13 (0.53 − 2.44)	7.515670e−01			0.89 (0.40 − 1.96)	0.7689527547	
Small intestine cancer	172967(88)		0.72 (0.26 − 1.95)	5.142164e−01			0.53 (0.19 − 1.48)	0.2252898249	

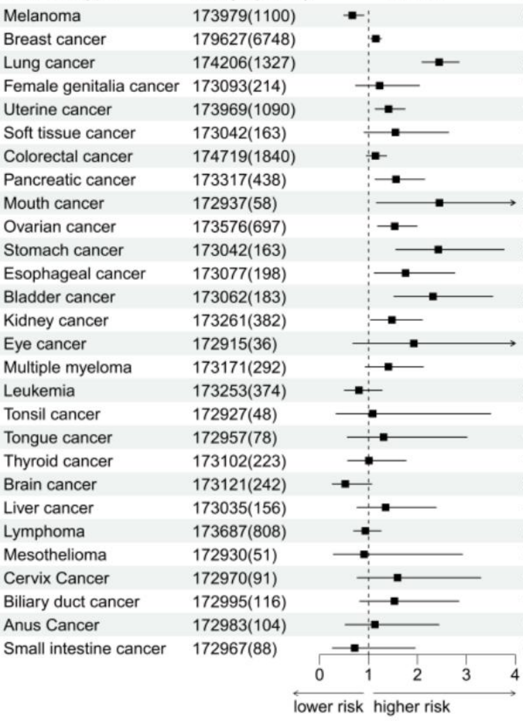

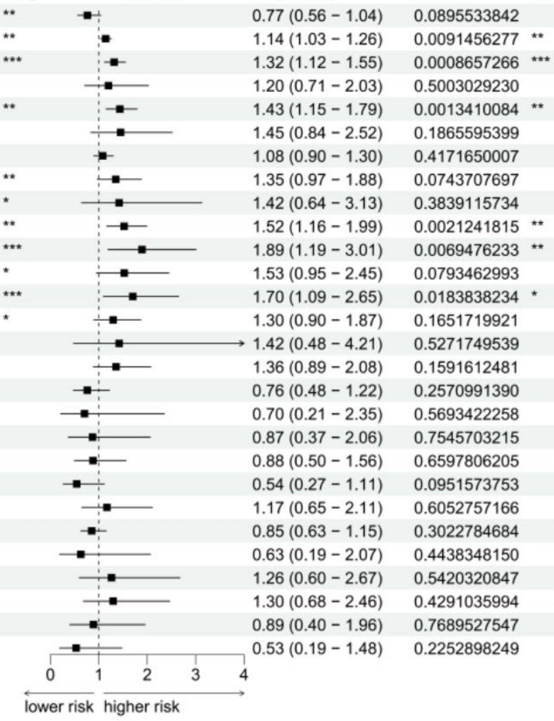

B

Outcome Type	Total(Exposed)	Model1	HR(95%CI)	Pvalue	Psig	Model2	HR(95%CI)	Pvalue	Psig
Melanoma	173979(1100)		0.66 (0.46 − 0.95)	2.622673e−02	*		0.71 (0.49 − 1.03)	0.07483765	
Breast cancer	179627(6748)		1.01 (0.89 − 1.14)	8.893082e−01			1.00 (0.89 − 1.13)	0.97878870	
Lung cancer	174206(1327)		1.63 (1.31 − 2.03)	1.451916e−05	***		1.11 (0.89 − 1.39)	0.34632214	
Female genitalia cancer	173093(214)		0.45 (0.17 − 1.22)	1.185158e−01			0.44 (0.16 − 1.19)	0.10704522	
Uterine cancer	173969(1090)		1.10 (0.83 − 1.47)	4.955467e−01			1.10 (0.83 − 1.47)	0.50236951	
Soft tissue cancer	173042(163)		0.60 (0.22 − 1.62)	3.155747e−01			0.57 (0.21 − 1.55)	0.27096286	
Colorectal cancer	174719(1840)		1.01 (0.80 − 1.27)	9.361269e−01			0.98 (0.78 − 1.24)	0.86567601	
Pancreatic cancer	173317(438)		1.19 (0.77 − 1.85)	4.294105e−01			1.11 (0.71 − 1.73)	0.64436579	
Mouth cancer	172937(58)		2.28 (0.91 − 5.71)	7.752484e−02			1.65 (0.65 − 4.18)	0.29564783	
Ovarian cancer	173576(697)		1.11 (0.77 − 1.59)	5.754208e−01			1.11 (0.77 − 1.60)	0.57383165	
Stomach cancer	173042(163)		1.53 (0.81 − 2.90)	1.944261e−01			1.29 (0.68 − 2.47)	0.43915032	
Esophageal cancer	173077(198)		1.96 (1.16 − 3.31)	1.242703e−02	*		1.77 (1.04 − 3.02)	0.03530122	*
Bladder cancer	173062(183)		1.37 (0.72 − 2.59)	3.359047e−01			1.20 (0.63 − 2.29)	0.57668421	
Kidney cancer	173261(382)		1.32 (0.84 − 2.08)	2.219460e−01			1.20 (0.76 − 1.89)	0.42715487	
Eye cancer	172915(36)		1.42 (0.34 − 5.91)	6.297052e−01			1.28 (0.30 − 5.40)	0.73946897	
Multiple myeloma	173171(292)		1.00 (0.56 − 1.79)	9.920162e−01			1.00 (0.56 − 1.79)	0.99906935	
Leukemia	173253(374)		1.07 (0.65 − 1.77)	7.920493e−01			1.03 (0.62 − 1.70)	0.91529391	
Tonsil cancer	172927(48)		1.04 (0.25 − 4.30)	9.532240e−01			0.80 (0.19 − 3.33)	0.75511471	
Tongue cancer	172957(78)		1.97 (0.86 − 4.54)	1.099768e−01			1.70 (0.73 − 3.95)	0.22212923	
Thyroid cancer	173102(223)		0.99 (0.51 − 1.93)	9.717092e−01			0.90 (0.46 − 1.76)	0.75847627	
Brain cancer	173121(242)		1.59 (0.94 − 2.67)	8.379158e−02			1.60 (0.94 − 2.72)	0.08040757	
Liver cancer	173035(156)		2.16 (1.22 − 3.81)	7.918112e−03	**		1.85 (1.04 − 3.30)	0.03586002	*
Lymphoma	173687(808)		1.14 (0.82 − 1.59)	4.291832e−01			1.07 (0.76 − 1.49)	0.70724594	
Mesothelioma	172930(51)		2.04 (0.73 − 5.66)	1.715847e−01			1.78 (0.63 − 5.00)	0.27284110	
Cervix Cancer	172970(91)		1.08 (0.40 − 2.95)	8.781032e−01			0.95 (0.35 − 2.62)	0.92550329	
Biliary duct cancer	172995(116)		0.63 (0.20 − 1.97)	4.233401e−01			0.55 (0.17 − 1.73)	0.30606501	
Anus Cancer	172983(104)		1.22 (0.50 − 2.99)	6.653036e−01			1.09 (0.44 − 2.70)	0.84857783	
Small intestine cancer	172967(88)		1.12 (0.41 − 3.05)	8.247200e−01			0.97 (0.35 − 2.66)	0.94819068	

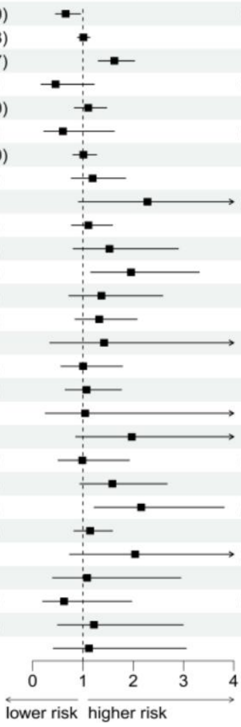

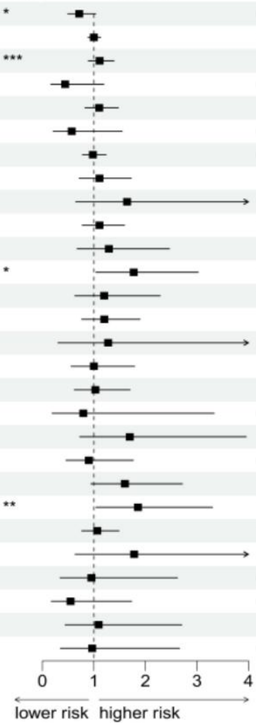

Panel (A) illustrates the CSHR of SI on specific cancer types in females using model1 and model2, (B) illustrates the CSHR of loneliness on specific cancer types in females using model1 and model2. CSHR and 95%CI were estimated by cause-specific Cox proportional hazards model using age as the underlying time variable. The model1 was adjusted for age, sex, and race/ethnicity, the model2 was further adjusted for assessment center, employment, college/university degree, sun exposure time, Townsend deprivation score, smoking status, and alcohol use. Psig was determined based on P value, with * for $P < 0.05$, ** for $P < 0.01$, *** for $P < 0.001$. Abbreviation: CSHR, Cause-Specific Hazard Ratio; sHR, Subdistribution Hazard Ratio; HR, Hazard Ratio; CI, Confidence Interval; SI, Social Isolation.

eFigure7. Associations of SI, Loneliness with Organ-Specific Cancer Risks in Male Participants (Model 1 and Model 2).

A

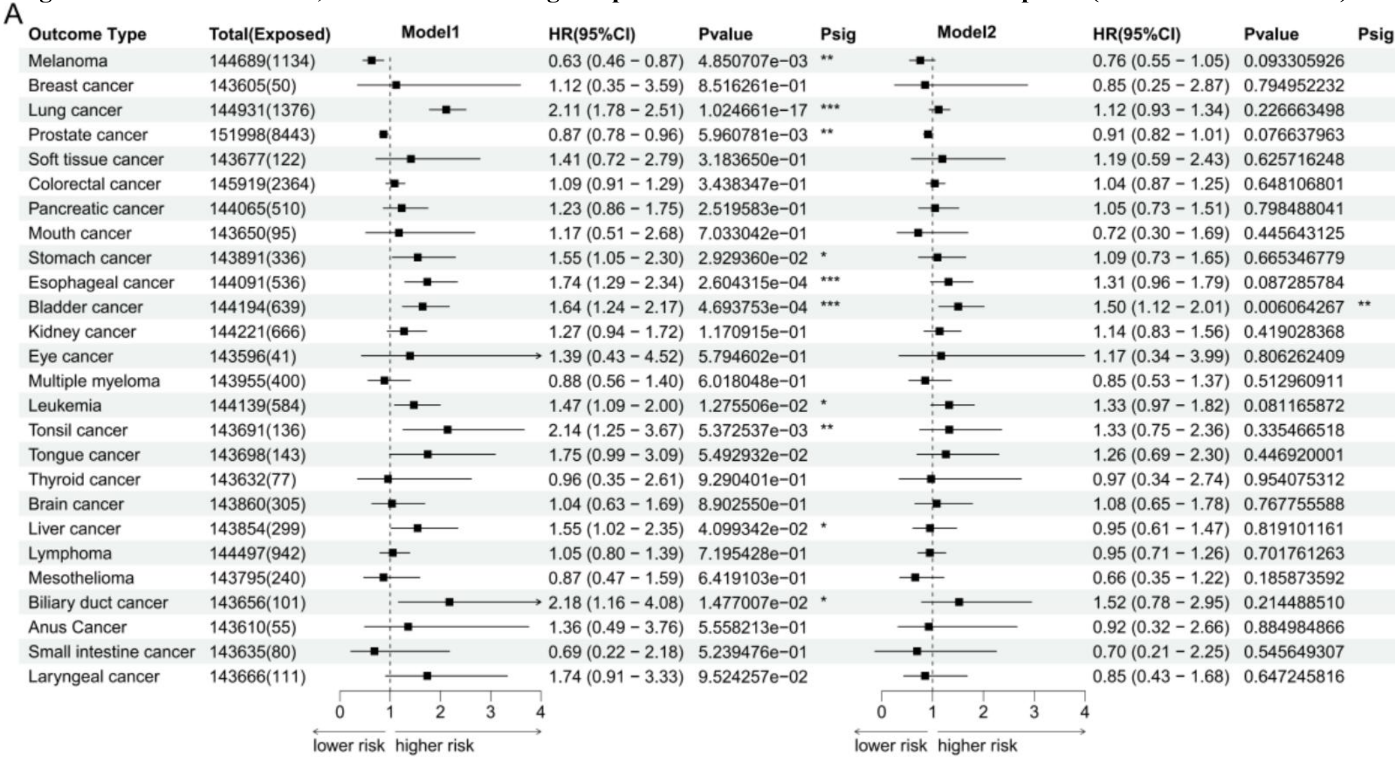

Outcome Type	Total(Exposed)	Model1 HR(95%CI)	Pvalue	Psig	Model2 HR(95%CI)	Pvalue	Psig
Melanoma	144689(1134)	0.63 (0.46 − 0.87)	4.850707e−03	**	0.76 (0.55 − 1.05)	0.093305926	
Breast cancer	143605(50)	1.12 (0.35 − 3.59)	8.516261e−01		0.85 (0.25 − 2.87)	0.794952232	
Lung cancer	144931(1376)	2.11 (1.78 − 2.51)	1.024661e−17	***	1.12 (0.93 − 1.34)	0.226663498	
Prostate cancer	151998(8443)	0.87 (0.78 − 0.96)	5.960781e−03	**	0.91 (0.82 − 1.01)	0.076637963	
Soft tissue cancer	143677(122)	1.41 (0.72 − 2.79)	3.183650e−01		1.19 (0.59 − 2.43)	0.625716248	
Colorectal cancer	145919(2364)	1.09 (0.91 − 1.29)	3.438347e−01		1.04 (0.87 − 1.25)	0.648106801	
Pancreatic cancer	144065(510)	1.23 (0.86 − 1.75)	2.519583e−01		1.05 (0.73 − 1.51)	0.798488041	
Mouth cancer	143650(95)	1.17 (0.51 − 2.68)	7.033042e−01		0.72 (0.30 − 1.69)	0.445643125	
Stomach cancer	143891(336)	1.55 (1.05 − 2.30)	2.929360e−02	*	1.09 (0.73 − 1.65)	0.665346779	
Esophageal cancer	144091(536)	1.74 (1.29 − 2.34)	2.604315e−04	***	1.31 (0.96 − 1.79)	0.087285784	
Bladder cancer	144194(639)	1.64 (1.24 − 2.17)	4.693753e−04	***	1.50 (1.12 − 2.01)	0.006064267	**
Kidney cancer	144221(666)	1.27 (0.94 − 1.72)	1.170915e−01		1.14 (0.83 − 1.56)	0.419028368	
Eye cancer	143596(41)	1.39 (0.43 − 4.52)	5.794602e−01		1.17 (0.34 − 3.99)	0.806262409	
Multiple myeloma	143955(400)	0.88 (0.56 − 1.40)	6.018048e−01		0.85 (0.53 − 1.37)	0.512960911	
Leukemia	144139(584)	1.47 (1.09 − 2.00)	1.275506e−02	*	1.33 (0.97 − 1.82)	0.081165872	
Tonsil cancer	143691(136)	2.14 (1.25 − 3.67)	5.372537e−03	**	1.33 (0.75 − 2.36)	0.335466518	
Tongue cancer	143698(143)	1.75 (0.99 − 3.09)	5.492932e−02		1.26 (0.69 − 2.30)	0.446920001	
Thyroid cancer	143632(77)	0.96 (0.35 − 2.61)	9.290401e−01		0.97 (0.34 − 2.74)	0.954075312	
Brain cancer	143860(305)	1.04 (0.63 − 1.69)	8.902550e−01		1.08 (0.65 − 1.78)	0.767755588	
Liver cancer	143854(299)	1.55 (1.02 − 2.35)	4.099342e−02	*	0.95 (0.61 − 1.47)	0.819101161	
Lymphoma	144497(942)	1.05 (0.80 − 1.39)	7.195428e−01		0.95 (0.71 − 1.26)	0.701761263	
Mesothelioma	143795(240)	0.87 (0.47 − 1.59)	6.419103e−01		0.66 (0.35 − 1.22)	0.185873592	
Biliary duct cancer	143656(101)	2.18 (1.16 − 4.08)	1.477007e−02	*	1.52 (0.78 − 2.95)	0.214488510	
Anus Cancer	143610(55)	1.36 (0.49 − 3.76)	5.558213e−01		0.92 (0.32 − 2.66)	0.884984866	
Small intestine cancer	143635(80)	0.69 (0.22 − 2.18)	5.239476e−01		0.70 (0.21 − 2.25)	0.545649307	
Laryngeal cancer	143666(111)	1.74 (0.91 − 3.33)	9.524257e−02		0.85 (0.43 − 1.68)	0.647245816	

B

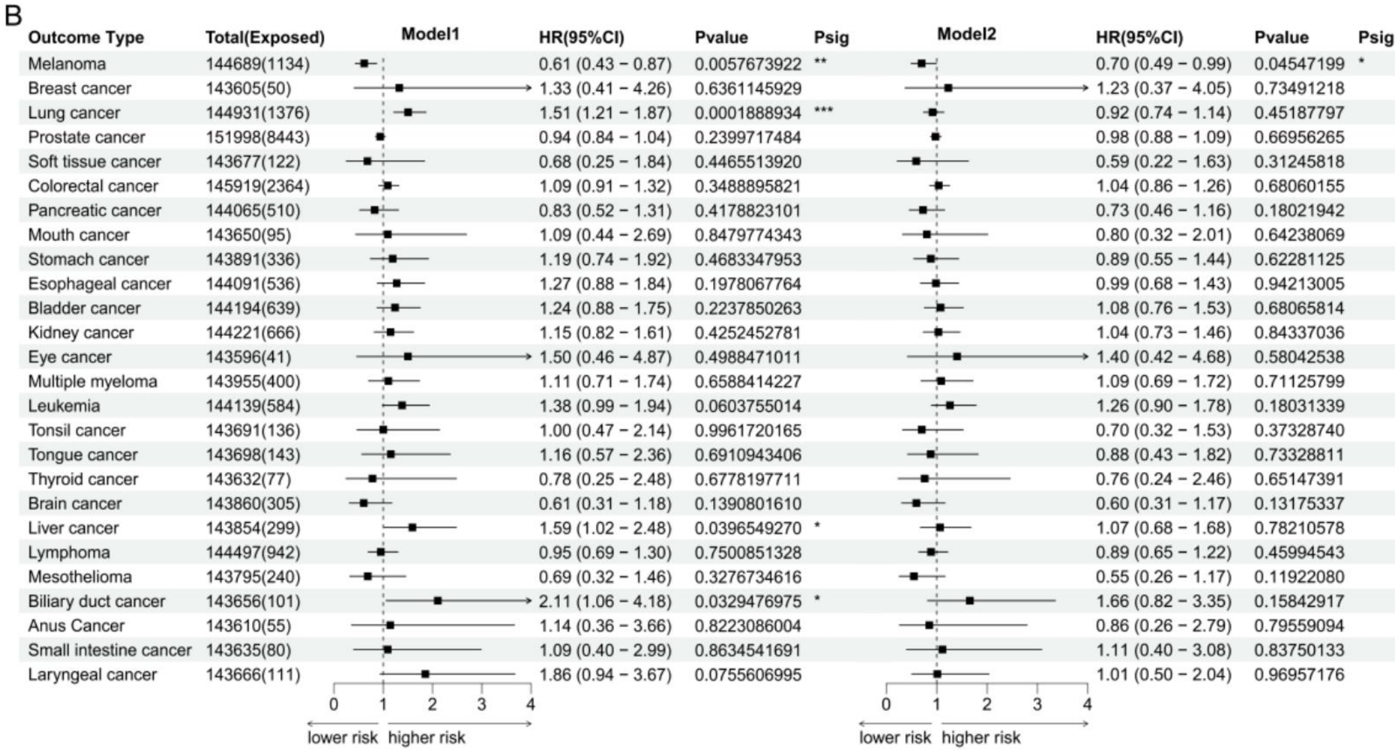

Outcome Type	Total(Exposed)	Model1 HR(95%CI)	Pvalue	Psig	Model2 HR(95%CI)	Pvalue	Psig
Melanoma	144689(1134)	0.61 (0.43 − 0.87)	0.0057673922	**	0.70 (0.49 − 0.99)	0.04547199	*
Breast cancer	143605(50)	1.33 (0.41 − 4.26)	0.6361145929		1.23 (0.37 − 4.05)	0.73491218	
Lung cancer	144931(1376)	1.51 (1.21 − 1.87)	0.0001888934	***	0.92 (0.74 − 1.14)	0.45187797	
Prostate cancer	151998(8443)	0.94 (0.84 − 1.04)	0.2399717484		0.98 (0.88 − 1.09)	0.66956265	
Soft tissue cancer	143677(122)	0.68 (0.25 − 1.84)	0.4465513920		0.59 (0.22 − 1.63)	0.31245818	
Colorectal cancer	145919(2364)	1.09 (0.91 − 1.32)	0.3488895821		1.04 (0.86 − 1.26)	0.68060155	
Pancreatic cancer	144065(510)	0.83 (0.52 − 1.31)	0.4178823101		0.73 (0.46 − 1.16)	0.18021942	
Mouth cancer	143650(95)	1.09 (0.44 − 2.69)	0.8479774343		0.80 (0.32 − 2.01)	0.64238069	
Stomach cancer	143891(336)	1.19 (0.74 − 1.92)	0.4683347953		0.89 (0.55 − 1.44)	0.62281125	
Esophageal cancer	144091(536)	1.27 (0.88 − 1.84)	0.1978067764		0.99 (0.68 − 1.43)	0.94213005	
Bladder cancer	144194(639)	1.24 (0.88 − 1.75)	0.2237850263		1.08 (0.76 − 1.53)	0.68065814	
Kidney cancer	144221(666)	1.15 (0.82 − 1.61)	0.4252452781		1.04 (0.73 − 1.46)	0.84337036	
Eye cancer	143596(41)	1.50 (0.46 − 4.87)	0.4988471011		1.40 (0.42 − 4.68)	0.58042538	
Multiple myeloma	143955(400)	1.11 (0.71 − 1.74)	0.6588414227		1.09 (0.69 − 1.72)	0.71125799	
Leukemia	144139(584)	1.38 (0.99 − 1.94)	0.0603755014		1.26 (0.90 − 1.78)	0.18031339	
Tonsil cancer	143691(136)	1.00 (0.47 − 2.14)	0.9961720165		0.70 (0.32 − 1.53)	0.37328740	
Tongue cancer	143698(143)	1.16 (0.57 − 2.36)	0.6910943406		0.88 (0.43 − 1.82)	0.73328811	
Thyroid cancer	143632(77)	0.78 (0.25 − 2.48)	0.6778197711		0.76 (0.24 − 2.46)	0.65147391	
Brain cancer	143860(305)	0.61 (0.31 − 1.18)	0.1390801610		0.60 (0.31 − 1.17)	0.13175337	
Liver cancer	143854(299)	1.59 (1.02 − 2.48)	0.0396549270	*	1.07 (0.68 − 1.68)	0.78210578	
Lymphoma	144497(942)	0.95 (0.69 − 1.30)	0.7500851328		0.89 (0.65 − 1.22)	0.45994543	
Mesothelioma	143795(240)	0.69 (0.32 − 1.46)	0.3276734616		0.55 (0.26 − 1.17)	0.11922080	
Biliary duct cancer	143656(101)	2.11 (1.06 − 4.18)	0.0329476975	*	1.66 (0.82 − 3.35)	0.15842917	
Anus Cancer	143610(55)	1.14 (0.36 − 3.66)	0.8223086004		0.86 (0.26 − 2.79)	0.79559094	
Small intestine cancer	143635(80)	1.09 (0.40 − 2.99)	0.8634541691		1.11 (0.40 − 3.08)	0.83750133	
Laryngeal cancer	143666(111)	1.86 (0.94 − 3.67)	0.0755606995		1.01 (0.50 − 2.04)	0.96957176	

Panel (A) illustrates the CSHR of SI on specific cancer types in males using model1 and model2, (B) illustrates the CSHR of loneliness on specific cancer types in males using model1 and model2. CSHR and 95%CI were estimated by cause-specific Cox proportional hazards model using age as the underlying time variable. The model1 was adjusted for age, sex, and race/ethnicity, the model2 was further adjusted for assessment center, employment, college/university degree, sun exposure time, Townsend deprivation score, smoking status, and alcohol use. Psig was determined based on P value, with * for P < 0.05, ** for P < 0.01, *** for P < 0.001. Abbreviation: CSHR, Cause-Specific Hazard Ratio; sHR, Subdistribution Hazard Ratio; HR, Hazard Ratio; CI, Confidence Interval; SI, Social Isolation.

eFigure8. Associations of SI and Loneliness with Organ-Specific Cancer Risk by Cancer Type and Sex (Model3).

A

Outcome Type	Total(Exposed)	Social Isolation HR(95%CI)	Pvalue	Psig	Loneliness HR(95%CI)	Pvalue	Psig
Melanoma	144689(1134)	0.77 (0.55 − 1.06)	0.109269628		0.72 (0.50 − 1.02)	0.06263246	
Breast cancer	143605(50)	0.88 (0.26 − 2.98)	0.841940572		1.30 (0.38 − 4.40)	0.67227296	
Lung cancer	144931(1376)	1.10 (0.92 − 1.32)	0.315105548		0.85 (0.68 − 1.07)	0.16610817	
Prostate cancer	151998(8443)	0.92 (0.83 − 1.02)	0.097763736		1.00 (0.89 − 1.11)	0.96303667	
Soft tissue cancer	143677(122)	1.17 (0.57 − 2.38)	0.669216265		0.58 (0.21 − 1.61)	0.29867701	
Colorectal cancer	145919(2364)	1.04 (0.87 − 1.24)	0.689294287		1.04 (0.86 − 1.25)	0.71582257	
Pancreatic cancer	144065(510)	1.05 (0.73 − 1.51)	0.797613103		0.71 (0.44 − 1.13)	0.14580877	
Mouth cancer	143650(95)	0.68 (0.29 − 1.62)	0.387683122		0.76 (0.30 − 1.92)	0.55854871	
Stomach cancer	143891(336)	1.08 (0.72 − 1.63)	0.711626720		0.85 (0.52 − 1.38)	0.50797014	
Esophageal cancer	144091(536)	1.28 (0.94 − 1.75)	0.117807774		0.92 (0.63 − 1.35)	0.67251705	
Bladder cancer	144194(639)	1.50 (1.12 − 2.01)	0.006104089	**	1.09 (0.76 − 1.56)	0.63090838	
Kidney cancer	144221(666)	1.11 (0.81 − 1.53)	0.499062194		0.97 (0.68 − 1.38)	0.85166829	
Eye cancer	143596(41)	1.18 (0.35 − 4.04)	0.786881444		1.50 (0.44 − 5.07)	0.51535692	
Multiple myeloma	143955(400)	0.85 (0.53 − 1.37)	0.511826862		1.11 (0.70 − 1.76)	0.65992364	
Leukemia	144139(584)	1.32 (0.96 − 1.82)	0.084841298		1.24 (0.88 − 1.76)	0.22247100	
Tonsil cancer	143691(136)	1.34 (0.75 − 2.39)	0.317981918		0.76 (0.35 − 1.67)	0.49387660	
Tongue cancer	143698(143)	1.22 (0.67 − 2.23)	0.517452731		0.80 (0.38 − 1.68)	0.55874288	
Thyroid cancer	143632(77)	0.97 (0.34 − 2.73)	0.946519429		0.80 (0.25 − 2.59)	0.70983950	
Brain cancer	143860(305)	1.11 (0.67 − 1.83)	0.688668661		0.66 (0.34 − 1.29)	0.22549544	
Liver cancer	143854(299)	0.92 (0.59 − 1.42)	0.706139771		0.97 (0.61 − 1.54)	0.90923254	
Lymphoma	144497(942)	0.95 (0.72 − 1.27)	0.741518098		0.88 (0.64 − 1.21)	0.42643493	
Mesothelioma	143795(240)	0.65 (0.35 − 1.20)	0.168929015		0.52 (0.24 − 1.13)	0.09817447	
Biliary duct cancer	143656(101)	1.53 (0.79 − 2.96)	0.208926477		1.76 (0.86 − 3.58)	0.12130900	
Anus Cancer	143610(55)	0.91 (0.31 − 2.61)	0.854052331		0.93 (0.28 − 3.05)	0.90144817	
Small intestine cancer	143635(80)	0.66 (0.20 − 2.13)	0.484368502		0.93 (0.33 − 2.62)	0.88500833	
Laryngeal cancer	143666(111)	0.88 (0.44 − 1.73)	0.702948175		1.08 (0.53 − 2.20)	0.82264221	

B

Outcome Type	Total(Exposed)	Social Isolation HR(95%CI)	Pvalue	Psig	Loneliness HR(95%CI)	Pvalue	Psig
Melanoma	173979(1100)	0.78 (0.57 − 1.06)	0.111877810		0.76 (0.53 − 1.11)	0.15449236	
Breast cancer	179627(6748)	1.13 (1.03 − 1.25)	0.012795491	*	1.00 (0.89 − 1.13)	0.97736888	
Lung cancer	174206(1327)	1.29 (1.09 − 1.51)	0.002508865	**	1.06 (0.85 − 1.33)	0.59446248	
Female genitalia cancer	173093(214)	1.19 (0.70 − 2.01)	0.517628465		0.43 (0.16 − 1.15)	0.09339111	
Uterine cancer	173969(1090)	1.36 (1.09 − 1.70)	0.006180872	**	1.06 (0.79 − 1.42)	0.70293647	
Soft tissue cancer	173042(163)	1.38 (0.79 − 2.40)	0.251878292		0.50 (0.18 − 1.36)	0.17592801	
Colorectal cancer	174719(1840)	1.09 (0.90 − 1.31)	0.383083161		1.01 (0.80 − 1.28)	0.92571495	
Pancreatic cancer	173317(438)	1.34 (0.96 − 1.86)	0.086826094		1.08 (0.69 − 1.69)	0.74327489	
Mouth cancer	172937(58)	1.48 (0.67 − 3.27)	0.330121178		1.89 (0.73 − 4.87)	0.18631177	
Ovarian cancer	173576(697)	1.52 (1.16 − 1.99)	0.002269382	**	1.10 (0.76 − 1.58)	0.62108857	
Stomach cancer	173042(163)	1.84 (1.16 − 2.93)	0.009919714	**	1.23 (0.64 − 2.37)	0.54275931	
Esophageal cancer	173077(198)	1.47 (0.92 − 2.36)	0.110683164		1.66 (0.97 − 2.87)	0.06601904	
Bladder cancer	173062(183)	1.68 (1.08 − 2.62)	0.020983195	*	1.23 (0.64 − 2.35)	0.53966751	
Kidney cancer	173261(382)	1.23 (0.86 − 1.78)	0.260992381		1.12 (0.71 − 1.78)	0.62407325	
Eye cancer	172915(36)	1.35 (0.46 − 4.02)	0.585271423		1.12 (0.26 − 4.85)	0.88446692	
Multiple myeloma	173171(292)	1.35 (0.88 − 2.06)	0.168910011		1.03 (0.57 − 1.85)	0.92854484	
Leukemia	173253(374)	0.74 (0.46 − 1.19)	0.213835939		0.99 (0.60 − 1.65)	0.97764962	
Tonsil cancer	172927(48)	0.71 (0.21 − 2.38)	0.582884147		0.87 (0.20 − 3.68)	0.84451261	
Tongue cancer	172957(78)	0.83 (0.35 − 1.98)	0.680313643		1.55 (0.65 − 3.70)	0.32010219	
Thyroid cancer	173102(223)	0.85 (0.48 − 1.52)	0.591427524		0.87 (0.44 − 1.72)	0.69219292	
Brain cancer	173121(242)	0.54 (0.26 − 1.10)	0.087727154		1.55 (0.90 − 2.64)	0.11202496	
Liver cancer	173035(156)	1.10 (0.61 − 1.99)	0.740626888		1.65 (0.91 − 2.97)	0.09727105	
Lymphoma	173687(808)	0.84 (0.62 − 1.14)	0.256628728		1.00 (0.71 − 1.40)	0.99585632	
Mesothelioma	172930(51)	0.63 (0.19 − 2.08)	0.449744909		1.75 (0.61 − 5.01)	0.29720941	
Cervix Cancer	172970(91)	1.30 (0.62 − 2.76)	0.489332340		1.08 (0.39 − 3.00)	0.88256447	
Biliary duct cancer	172995(116)	1.27 (0.67 − 2.41)	0.471217167		0.49 (0.15 − 1.56)	0.22799305	
Anus Cancer	172983(104)	0.87 (0.39 − 1.92)	0.730440493		1.03 (0.41 − 2.58)	0.95526349	
Small intestine cancer	172967(88)	0.52 (0.19 − 1.46)	0.214794989		0.94 (0.34 − 2.62)	0.90656637	

Panel (A) illustrates the CSHR of SI and loneliness on specific cancer types of males, (B) illustrates the CSHR of SI and loneliness on specific cancer types of females. CSHR and 95%CI were estimated by cause-specific Cox proportional hazards model using age as the underlying time variable. The model was adjusted for age, race, assessment center, Employment, college/university degree, sun exposure time, Townsend deprivation score, smoking status, alcohol use, BMI, grip strength, family history of cancer, MAP, overall health rating, healthy diet score, healthy sleep score and depress mood. Psig was determined based on P value, with * for $P < 0.05$, ** for $P < 0.01$, *** for $P < 0.001$. Abbreviation: CSHR, Cause-Specific Hazard Ratio; sHR, Subdistribution Hazard Ratio; HR, Hazard Ratio; CI, Confidence Interval; SI, Social Isolation; BMI, Body Mass Index; MAP, Mean Arterial Pressure.

eFigure9. Mediation Effects of Hormone, Menopause Status and Inflammatory Markers on Cancer Risk in Participants Exposed to SI.

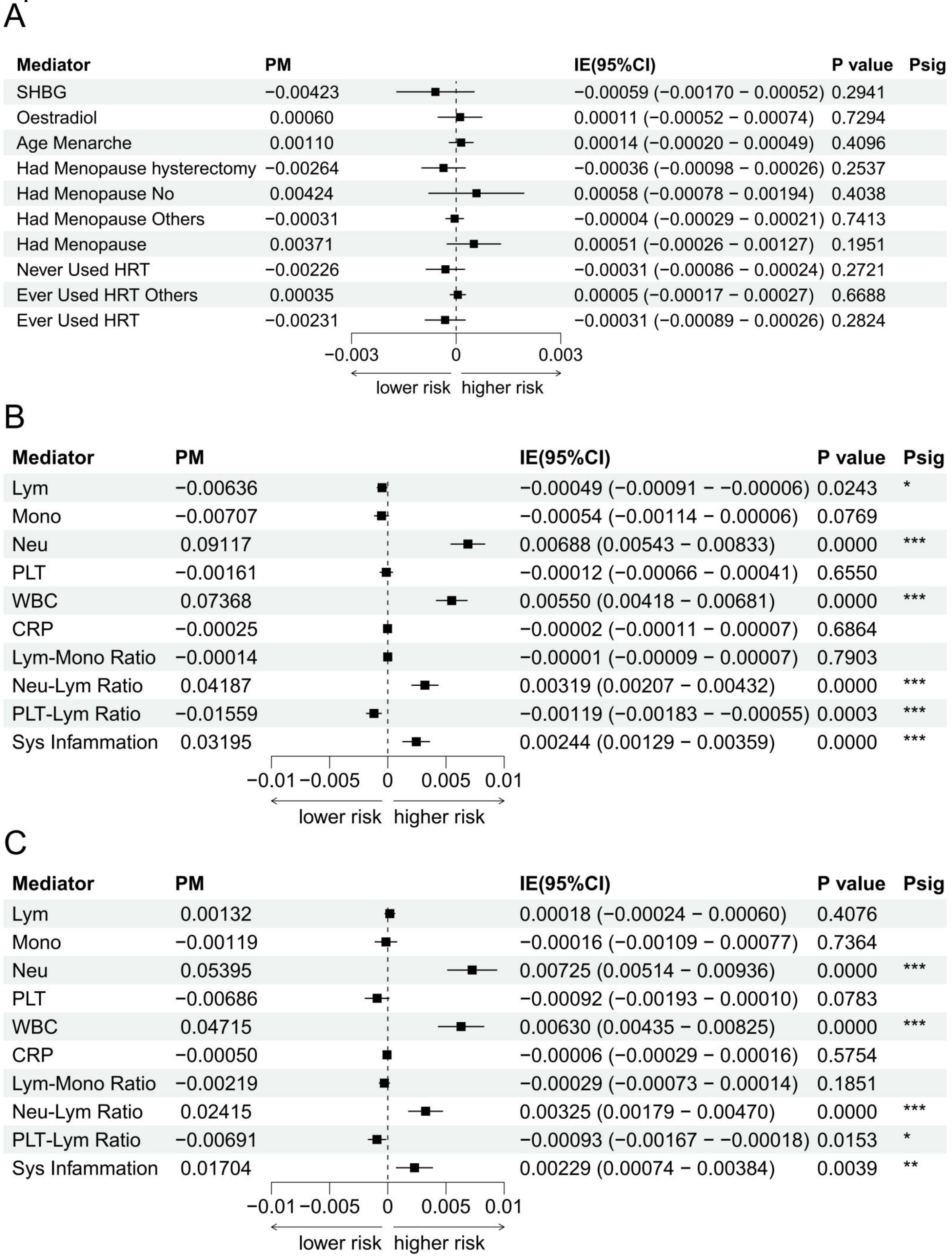

Panel (A) illustrates the mediation effect of hormone and menopause status in females, (B) illustrates the mediation effect of inflammatory markers in overall participants, (C) illustrates the mediation effect of inflammatory markers in female participants. Abbreviation: IE, Indirect Effect; CI, Confidence Interval; PM, Proportion Mediated; SHBG, Sex Hormone-Binding Globulin; HRT, Hormone Replacement Therapy; Lym, Lymphocytes; Mono, Monocytes; Neu, Neutrophils; PLT, Platelets; WBC, White Blood Cells; CRP, C-reactive Protein; Lym-mono Ratio, Lymphocyte to Monocyte Ratio; Neu-lym ratio, Neutrophil to Lymphocyte Ratio; PLT-lym Ratio, Platelet to Lymphocyte Ratio; SI, Social Isolation.

eFigure10. Mediation Effects of Hormone and Menopause Status on Specific Cancer Risk in Female Participants with SI Exposure.

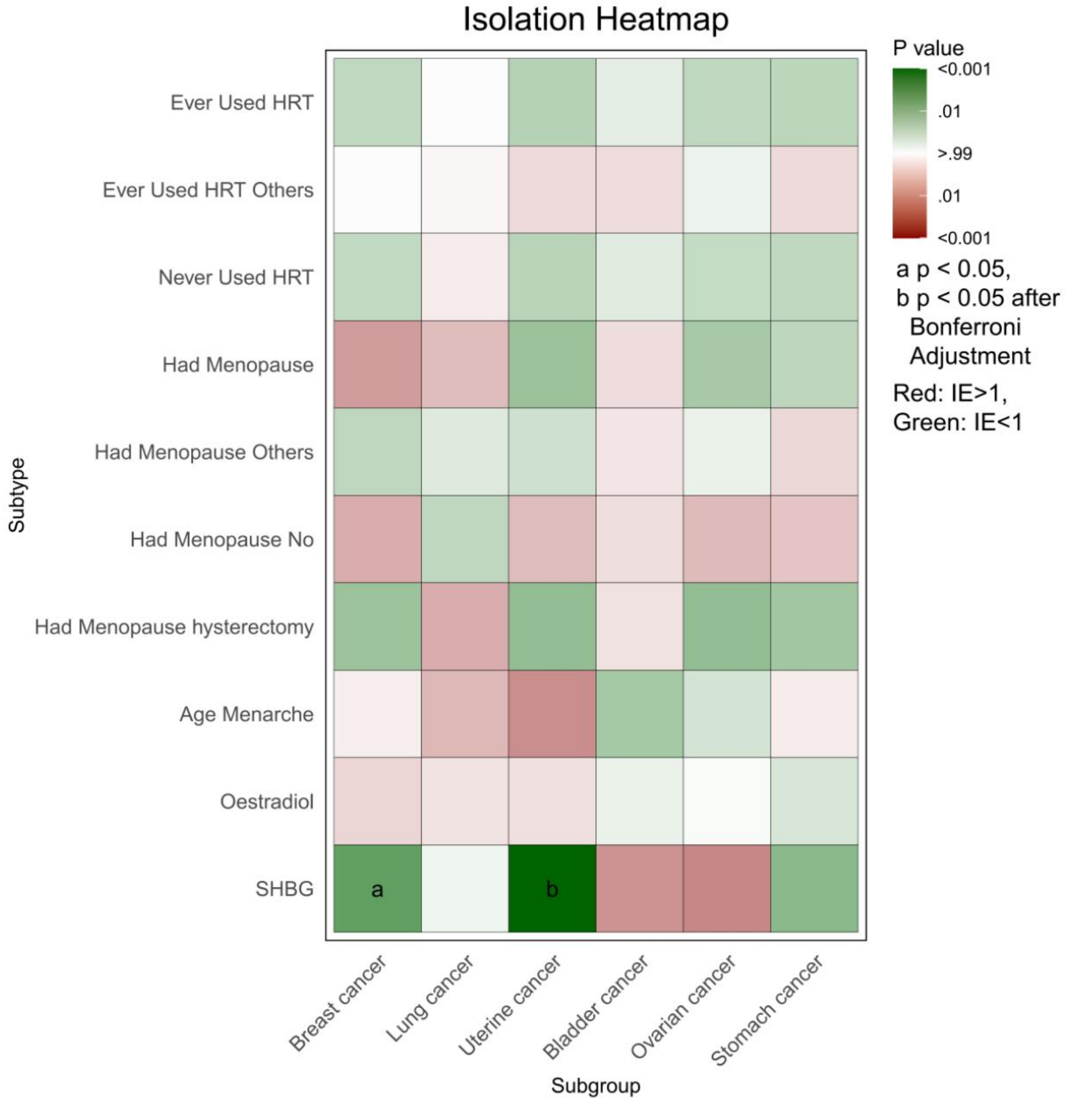

Heatmaps depicting the indirect effect of hormone levels and menopause status in mediating the relationship between SI and specific cancer risk in females. Color gradients represent P-values, with green indicating decreased risk (IE < 1) and red indicating increased risk (IE > 1). Significant associations are marked with "a" for P < 0.05 and "b" for P < 0.05 after bonferroni adjustment. Abbreviation：IE, Indirect Effect; CI, Confidence Interval; SHBG, Sex Hormone-Binding Globulin; HRT, Hormone Replacement Therapy.

eFigure11. Directed Acyclic Graph.

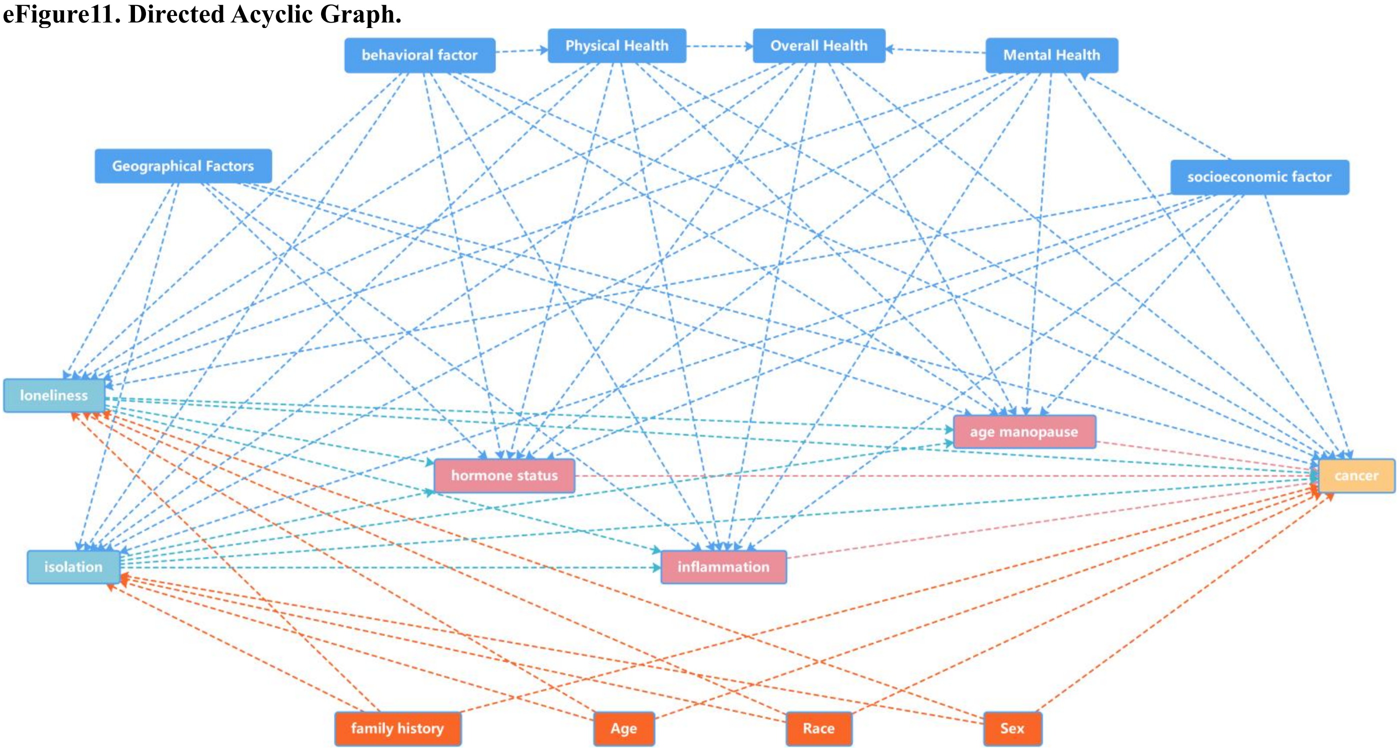

Directed Acyclic Graph illustrates the hypothesized relationships between variables and cancer risk. Nodes represent variables, arrows indicate directional relationships. Cerulean blue nodes represent confounders (modifiable factors), pink nodes represent mediators, orange nodes represent the outcome (cancer), red nodes represent unchangeable confounders (non-modifiable factors), and sky blue is also used for the exposure (isolation and loneliness).

eFigure12. Cumulative Incidence of Cancer by Social Isolation and Loneliness, Accounting for Competing Risks of Death, Over Up to 14 Years of Follow-up (Sensitive Analysis).

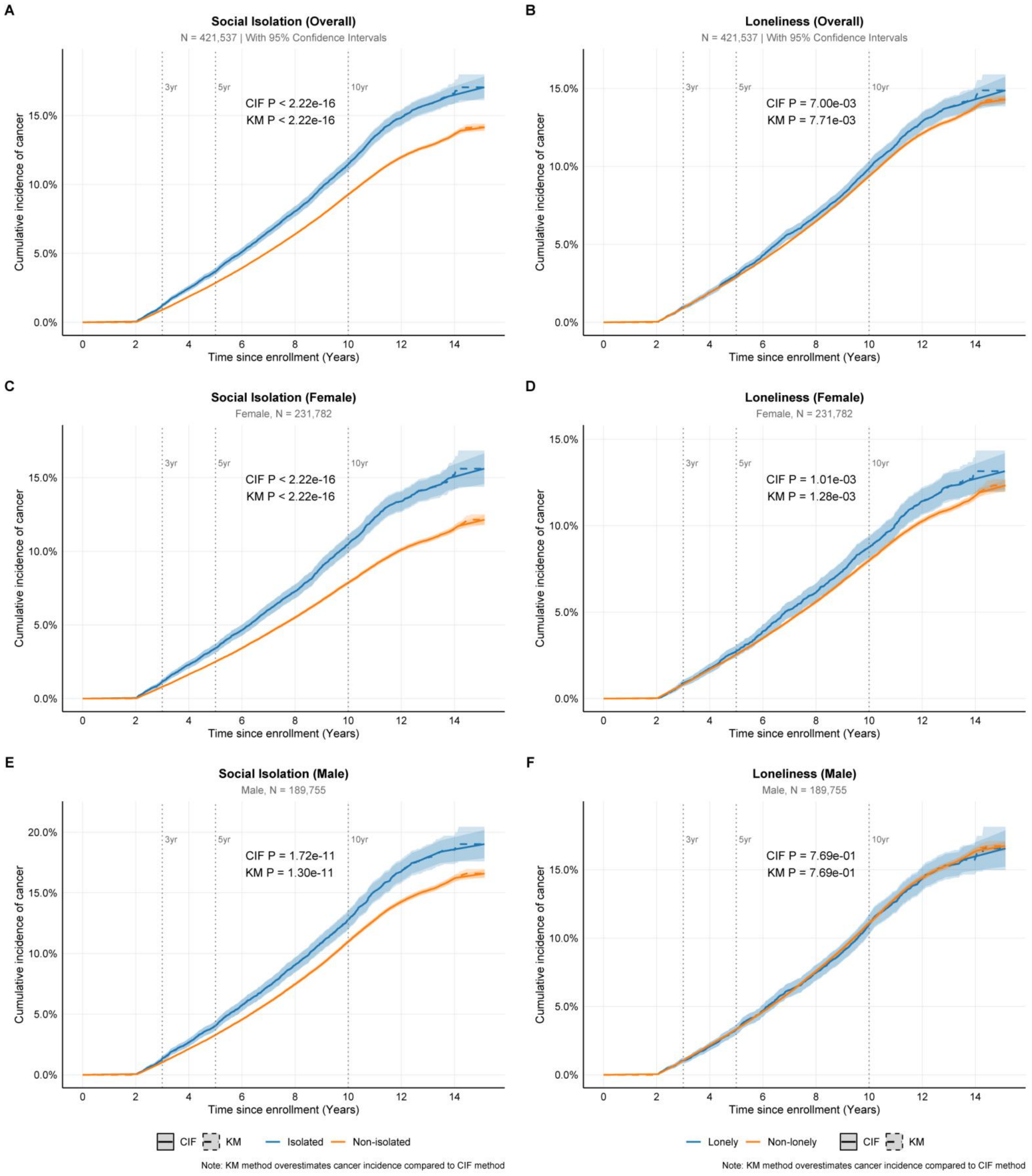

CIF curve (solid lines), and KM curve (dashed lines) illustrating estimates of association between SI or loneliness and cancer incidence in the overall population and stratified by sex. Panel (A) illustrates the effect of SI on survival in the overall population, (B) shows the effect of loneliness on survival in the overall population, (C) shows the effect of SI on survival in males, (D) shows the effect of loneliness on survival in males, (E) shows the effect of SI on survival in females, and (F) shows the effect of loneliness on survival in females. Shaded areas represent 95% confidence intervals. P-values (CIF and KM) for the difference between groups at 3, 5, and 10 years are annotated. Note: The KM method overestimates cancer incidence compared to the CIF method due to competing risks of death. Abbreviations: KM, Kaplan-Meier; CIF, Cumulative Incidence Functions; SI, Social Isolation.

eFigure13. Separate and Joint Association of SI and Loneliness with Long-term Risk of Cancer in Sensitive Analysis.

Group	Variable	N	Cases per 10000 Person Years	Model1 HR(95%CI)	Model2 HR(95%CI)	Model3 HR(95%CI)	PAF(95%CI)
Overall		421537	101				
Isolation							1.168 [0.691 − 1.625]<br>1.142 [0.738 − 1.532]
	Unexpose	396164	99	1.000 [Reference]<br>1.000 [Reference]	1.000 [Reference]<br>1.000 [Reference]	1 [reference]<br>1 [reference]	
	Expose	25373	124	1.228 [1.178−1.280]<br>1.240 [1.199−1.284]	1.116 [1.069−1.165]<br>1.111 [1.073−1.151]	1.108 [1.061−1.156]<br>1.103 [1.064−1.143]	
	P Value			<2.22e-16<br><2.22e-16	4.29e-08<br>5.11e-09	3.88e-07<br>8.09e-08	
Loneliness							−0.370 [−0.934 − 0.165]<br>−0.107 [−0.680 − 0.437]
	Unexpose	401659	100	1.000 [Reference]<br>1.000 [Reference]	1.000 [Reference]<br>1.000 [Reference]	1.000 [Reference]<br>1.000 [Reference]	
	Expose	19878	106	1.056 [1.004−1.110]<br>1.094 [1.041−1.149]	0.980 [0.931−1.032]<br>1.006 [0.957−1.058]	0.965 [0.916−1.016]<br>0.990 [0.941−1.042]	
	P Value			3.61e-02<br>3.95e-04	0.444<br>0.809	0.179<br>0.705	
	P for interaction			0.328<br>0.360	0.660<br>0.383	0.516<br>0.307	
Joint Effect							0.437 [0.203 − 0.667]<br>0.484 [0.265 − 0.699]
	Rank1	379793	99	1.000 [Reference]<br>1.000 [Reference]	1.000 [Reference]<br>1.000 [Reference]	1.000 [Reference]<br>1.000 [Reference]	
	Rank2	16371	100	1.051 [1.001−1.104]<br>1.053 [0.998−1.112]	0.974 [0.924−1.028]<br>0.985 [0.933−1.040]	0.949 [0.896−1.005]<br>0.970 [0.918−1.025]	0.346 [0.161 − 0.529]<br>0.377 [0.206 − 0.544]
	Rank3	21866	123	1.227 [1.179−1.276]<br>1.225 [1.183−1.268]	1.112 [1.065−1.161]<br>1.105 [1.065−1.145]	1.105 [1.056−1.157]<br>1.096 [1.056−1.137]	0.422 [0.196 − 0.644]<br>0.446 [0.244 − 0.644]
	Rank4	3507	134	1.363 [1.243−1.494]<br>1.359 [1.228−1.505]	1.114 [1.003−1.237]<br>1.145 [1.033−1.269]	1.095 [0.978−1.226]<br>1.128 [1.016−1.253]	0.437 [0.203 − 0.667]<br>0.484 [0.265 − 0.699]
	P trend			<2.22e-16<br><2.22e-16	7.21e-07<br>6.51e-07	1.42e-05<br>1.78e-05	

Model1: adjusted for age, sex, and race/ethnicity; Model 2: further adjusted for assessment center, employment, college/university degree, sun exposure time, socioeconomic status (Townsend deprivation score), smoking status, and alcohol use; Model 3: further adjusted for BMI (continuous), grip strength, family history of cancer, MAP, overall health rating, healthy diet score, healthy sleep score, and depressive mood. Abbreviations: HR, Hazard Ratio; CI, Confidence Interval; PAF, Population Attributable Fraction.

eFigure14. Cancer Risk Associated with SI and Loneliness, Stratified by Demographic and Lifestyle Factors in Sensitive Analysis.

			Social Isolation			Loneliness		
Variable	Count	Percent	HR(95%CI)	$P_{interaction}$	RERI(95%CI)	HR(95%CI)	$P_{interaction}$	RERI(95%CI)
Overall	421537	100	1.108(1.061 to 1.156)<br>1.103(1.064 to 1.143)			0.965(0.916 to 1.016)<br>0.990(0.941 to 1.042)		
Age Group				0.481<br>0.559			5.92e-05<br>4.00e-05	
0−49	105406	25.01	1.134(1.000 to 1.287)<br>1.136(1.007 to 1.281)			0.828(0.718 to 0.956)<br>0.829(0.715 to 0.962)		
50−59	144479	34.27	1.111(1.037 to 1.189)<br>1.111(1.047 to 1.179)		0.030(−0.048 to 0.115)<br>0.007(−0.017 to 0.031)	1.017(0.940 to 1.100)<br>1.019(0.941 to 1.104)		0.033(−0.058 to 0.133)<br>−0.002(−0.024 to 0.019)
60 And Older	171652	40.72	1.105(1.053 to 1.160)<br>1.103(1.050 to 1.160)		0.001(−0.071 to 0.079)<br>0.014(−0.011 to 0.040)	1.022(0.964 to 1.084)<br>1.023(0.963 to 1.087)		0.105(0.012 to 0.207)<br>−0.016(−0.043 to 0.011)
Sex				0.258<br>0.293			0.262<br>0.274	
Female	231782	54.98	1.143(1.085 to 1.205)<br>1.145(1.079 to 1.215)			1.015(0.950 to 1.085)<br>1.019(0.952 to 1.090)		
Male	189755	45.02	1.072(1.015 to 1.132)<br>1.080(1.020 to 1.144)		−0.041(−0.109 to 0.031)<br>0.055(0.026 to 0.084)	0.981(0.923 to 1.042)<br>0.987(0.926 to 1.052)		−0.049(−0.129 to 0.038)<br>0.015(−0.018 to 0.048)
Income				0.246<br>0.239			0.034<br>0.029	
Medium	189927	45.06	1.149(1.078 to 1.225)<br>1.149(1.068 to 1.237)			0.950(0.883 to 1.022)<br>0.954(0.885 to 1.029)		
High	100093	23.74	0.976(0.828 to 1.151)<br>0.970(0.822 to 1.145)		−0.136(−0.270 to 0.023)<br>0.080(−0.051 to 0.211)	0.978(0.852 to 1.123)<br>0.980(0.856 to 1.122)		−0.048(−0.176 to 0.100)<br>0.061(−0.046 to 0.168)
Low	77005	18.27	1.072(1.013 to 1.133)<br>1.077(1.012 to 1.147)		−0.014(−0.084 to 0.063)<br>0.041(−0.063 to 0.144)	1.054(0.980 to 1.132)<br>1.056(0.975 to 1.145)		0.142(0.044 to 0.249)<br>0.022(−0.064 to 0.109)
Unreported	54512	12.93	1.066(0.961 to 1.182)<br>1.064(0.960 to 1.178)		−0.009(−0.111 to 0.104)<br>0.103(0.005 to 0.202)	0.887(0.786 to 1.001)<br>0.891(0.789 to 1.007)		−0.090(−0.199 to 0.033)<br>0.069(−0.014 to 0.152)
Education				0.215<br>0.215			0.134<br>0.122	
College/University Degree	212937	50.51	1.109(1.057 to 1.163)<br>1.112(1.066 to 1.159)			0.998(0.944 to 1.056)<br>1.002(0.945 to 1.062)		
Not College Degree	208600	49.49	1.081(1.016 to 1.150)<br>1.085(1.025 to 1.149)		0.049(−0.028 to 0.132)<br>0.005(−0.010 to 0.020)	0.961(0.892 to 1.035)<br>0.966(0.897 to 1.039)		0.072(−0.021 to 0.174)<br>0.006(−0.007 to 0.019)
Employment				0.565<br>0.544			0.002<br>0.001	
Employed	254227	60.31	1.090(1.030 to 1.155)<br>1.092(1.036 to 1.151)			0.922(0.860 to 0.988)<br>0.927(0.867 to 0.992)		
Others	35579	8.44	1.066(0.960 to 1.184)<br>1.073(0.971 to 1.186)		0.017(−0.085 to 0.130)<br>0.015(0.007 to 0.023)	1.007(0.899 to 1.129)<br>1.016(0.896 to 1.152)		0.050(−0.067 to 0.183)<br>0.006(−0.001 to 0.012)
Retired	131731	31.25	1.107(1.045 to 1.173)<br>1.106(1.038 to 1.179)		0.033(−0.041 to 0.112)<br>0.008(0.004 to 0.013)	1.054(0.985 to 1.129)<br>1.054(0.991 to 1.121)		0.144(0.047 to 0.250)<br>0.010(0.006 to 0.014)
Smoking Status				0.022<br>0.010			0.731<br>0.737	
Current	42850	10.17	1.106(1.024 to 1.194)<br>1.121(1.038 to 1.210)			0.957(0.869 to 1.054)<br>0.963(0.870 to 1.067)		
Never	234447	55.62	1.099(1.035 to 1.167)<br>1.101(1.043 to 1.162)		−0.030(−0.100 to 0.046)<br>−0.068(−0.100 to −0.036)	0.998(0.930 to 1.070)<br>1.000(0.928 to 1.078)		−0.011(−0.095 to 0.081)<br>−0.002(−0.037 to 0.033)
Previous	144240	34.22	1.061(0.996 to 1.131)<br>1.061(0.984 to 1.144)		−0.062(−0.131 to 0.013)<br>−0.041(−0.063 to −0.019)	0.973(0.905 to 1.046)<br>0.978(0.913 to 1.048)		−0.019(−0.104 to 0.073)<br>0.010(−0.015 to 0.036)
Alcohol Status				0.944<br>0.907			0.163<br>0.139	
Current	390548	92.65	1.102(1.058 to 1.148)<br>1.106(1.058 to 1.155)			0.981(0.935 to 1.028)<br>0.984(0.936 to 1.035)		
Never	16891	4.01	1.079(0.921 to 1.264)<br>1.077(0.919 to 1.262)		−0.023(−0.165 to 0.142)<br>0.011(0.003 to 0.018)	0.945(0.768 to 1.164)<br>0.951(0.792 to 1.142)		−0.055(−0.231 to 0.162)<br>0.011(0.007 to 0.015)
Previous	14098	3.34	1.057(0.923 to 1.211)<br>1.065(0.931 to 1.219)		0.013(−0.113 to 0.157)<br>0.018(0.010 to 0.026)	1.080(0.912 to 1.280)<br>1.090(0.926 to 1.284)		0.176(−0.006 to 0.391)<br>0.010(0.006 to 0.015)

Axis: 0.7 1 1.3 — Lower risk / Higher risk

Legend: Cox Proportional Hazards Regression; Competing Risk Regression

This forest plot presents HR and 95%CI for cancer risk related to SI and loneliness, stratified by demographic and lifestyle factors. Subgroup analyses cover age, sex, income, education, employment, smoking, and alcohol use. Multiplicative interactions were assessed using P for interaction, while additive interactions were evaluated with RERI. Abbreviations: HR, Hazard Ratios, CI, Confidence Intervals, RERI, Relative Excess Risk Due to Interaction.

eFigure15. Associations of SI, Loneliness with Cancer Risk in Male Participants by Cancer Types (Sensitive Analysis).

A

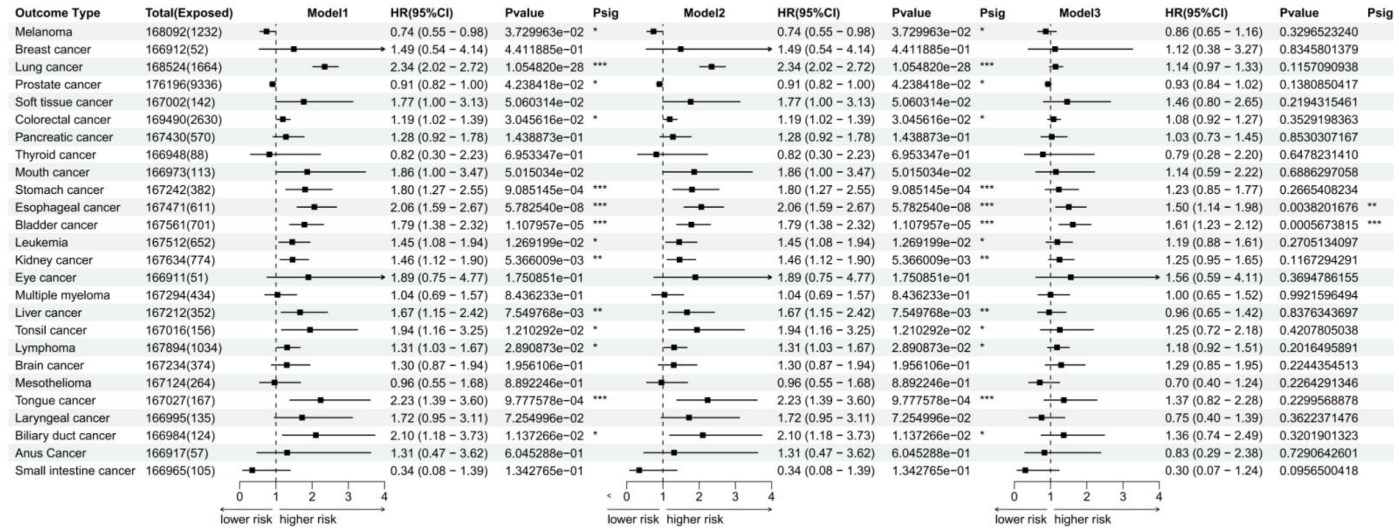

Outcome Type	Total(Exposed)	Model1	HR(95%CI)	Pvalue	Psig	Model2	HR(95%CI)	Pvalue	Psig	Model3	HR(95%CI)	Pvalue	Psig
Melanoma	168092(1232)		0.74 (0.55 − 0.98)	3.729963e−02	*		0.74 (0.55 − 0.98)	3.729963e−02	*		0.86 (0.65 − 1.16)	0.3296523240	
Breast cancer	166912(52)		1.49 (0.54 − 4.14)	4.411885e−01			1.49 (0.54 − 4.14)	4.411885e−01			1.12 (0.38 − 3.27)	0.8345801379	
Lung cancer	168524(1664)		2.34 (2.02 − 2.72)	1.054820e−28	***		2.34 (2.02 − 2.72)	1.054820e−28	***		1.14 (0.97 − 1.33)	0.1157090938	
Prostate cancer	176196(9336)		0.91 (0.82 − 1.00)	4.238418e−02	*		0.91 (0.82 − 1.00)	4.238418e−02	*		0.93 (0.84 − 1.02)	0.1380850417	
Soft tissue cancer	167002(142)		1.77 (1.00 − 3.13)	5.060314e−02			1.77 (1.00 − 3.13)	5.060314e−02			1.46 (0.80 − 2.65)	0.2194315461	
Colorectal cancer	169490(2630)		1.19 (1.02 − 1.39)	3.045616e−02	*		1.19 (1.02 − 1.39)	3.045616e−02	*		1.08 (0.92 − 1.27)	0.3529198363	
Pancreatic cancer	167430(570)		1.28 (0.92 − 1.78)	1.438873e−01			1.28 (0.92 − 1.78)	1.438873e−01			1.03 (0.73 − 1.45)	0.8530307167	
Thyroid cancer	166948(88)		0.82 (0.30 − 2.23)	6.953347e−01			0.82 (0.30 − 2.23)	6.953347e−01			0.79 (0.28 − 2.20)	0.6478231410	
Mouth cancer	166973(113)		1.86 (1.00 − 3.47)	5.015034e−02			1.86 (1.00 − 3.47)	5.015034e−02			1.14 (0.59 − 2.22)	0.6886297058	
Stomach cancer	167242(382)		1.80 (1.27 − 2.55)	9.085145e−04	***		1.80 (1.27 − 2.55)	9.085145e−04	***		1.23 (0.85 − 1.77)	0.2665408234	
Esophageal cancer	167471(611)		2.06 (1.59 − 2.67)	5.782540e−08	***		2.06 (1.59 − 2.67)	5.782540e−08	***		1.50 (1.14 − 1.98)	0.0038201676	**
Bladder cancer	167561(701)		1.79 (1.38 − 2.32)	1.107957e−05	***		1.79 (1.38 − 2.32)	1.107957e−05	***		1.61 (1.23 − 2.12)	0.0005673815	***
Leukemia	167512(652)		1.45 (1.08 − 1.94)	1.269199e−02	*		1.45 (1.08 − 1.94)	1.269199e−02	*		1.19 (0.88 − 1.61)	0.2705134097	
Kidney cancer	167634(774)		1.46 (1.12 − 1.90)	5.366009e−03	**		1.46 (1.12 − 1.90)	5.366009e−03	**		1.25 (0.95 − 1.65)	0.1167294291	
Eye cancer	166911(51)		1.89 (0.75 − 4.77)	1.750851e−01			1.89 (0.75 − 4.77)	1.750851e−01			1.56 (0.59 − 4.11)	0.3694786155	
Multiple myeloma	167294(434)		1.04 (0.69 − 1.57)	8.436233e−01			1.04 (0.69 − 1.57)	8.436233e−01			1.00 (0.65 − 1.52)	0.9921596494	
Liver cancer	167212(352)		1.67 (1.15 − 2.42)	7.549768e−03	**		1.67 (1.15 − 2.42)	7.549768e−03	**		0.96 (0.65 − 1.42)	0.8376343697	
Tonsil cancer	167016(156)		1.94 (1.16 − 3.25)	1.210292e−02	*		1.94 (1.16 − 3.25)	1.210292e−02	*		1.25 (0.72 − 2.18)	0.4207805038	
Lymphoma	167894(1034)		1.31 (1.03 − 1.67)	2.890873e−02	*		1.31 (1.03 − 1.67)	2.890873e−02	*		1.18 (0.92 − 1.51)	0.2016495891	
Brain cancer	167234(374)		1.30 (0.87 − 1.94)	1.956106e−01			1.30 (0.87 − 1.94)	1.956106e−01			1.29 (0.85 − 1.95)	0.2244354513	
Mesothelioma	167124(264)		0.96 (0.55 − 1.68)	8.892246e−01			0.96 (0.55 − 1.68)	8.892246e−01			0.70 (0.40 − 1.24)	0.2264291346	
Tongue cancer	167027(167)		2.23 (1.39 − 3.60)	9.777578e−04	***		2.23 (1.39 − 3.60)	9.777578e−04	***		1.37 (0.82 − 2.28)	0.2299568878	
Laryngeal cancer	166995(135)		1.72 (0.95 − 3.11)	7.254996e−02			1.72 (0.95 − 3.11)	7.254996e−02			0.75 (0.40 − 1.39)	0.3622371476	
Biliary duct cancer	166984(124)		2.10 (1.18 − 3.73)	1.137266e−02	*		2.10 (1.18 − 3.73)	1.137266e−02	*		1.36 (0.74 − 2.49)	0.3201901323	
Anus Cancer	166917(57)		1.31 (0.47 − 3.62)	6.045288e−01			1.31 (0.47 − 3.62)	6.045288e−01			0.83 (0.29 − 2.38)	0.7290642601	
Small intestine cancer	166965(105)		0.34 (0.08 − 1.39)	1.342765e−01			0.34 (0.08 − 1.39)	1.342765e−01			0.30 (0.07 − 1.24)	0.0956500418	

B

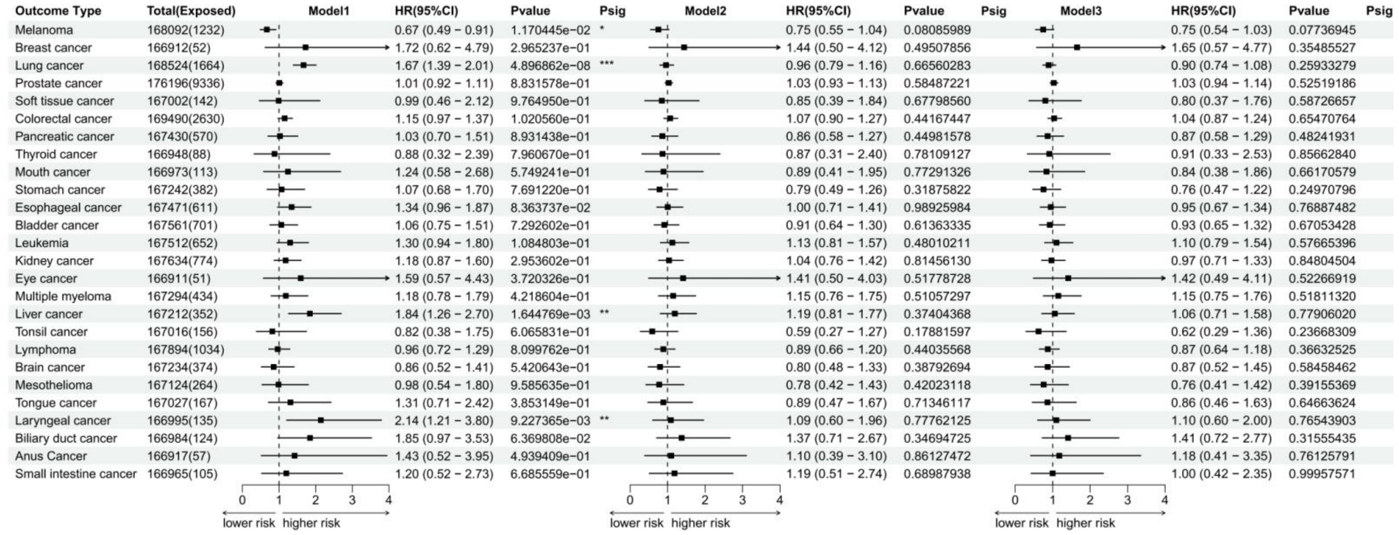

Outcome Type	Total(Exposed)	Model1	HR(95%CI)	Pvalue	Psig	Model2	HR(95%CI)	Pvalue	Psig	Model3	HR(95%CI)	Pvalue	Psig
Melanoma	168092(1232)		0.67 (0.49 − 0.91)	1.170445e−02	*		0.75 (0.55 − 1.04)	0.08085989			0.75 (0.54 − 1.03)	0.07736945	
Breast cancer	166912(52)		1.72 (0.62 − 4.79)	2.965237e−01			1.44 (0.50 − 4.12)	0.49507856			1.65 (0.57 − 4.77)	0.35485527	
Lung cancer	168524(1664)		1.67 (1.39 − 2.01)	4.896862e−08	***		0.96 (0.79 − 1.16)	0.66560283			0.90 (0.74 − 1.08)	0.25933279	
Prostate cancer	176196(9336)		1.01 (0.92 − 1.11)	8.831578e−01			1.03 (0.93 − 1.13)	0.58487221			1.03 (0.94 − 1.14)	0.52519186	
Soft tissue cancer	167002(142)		0.99 (0.46 − 2.12)	9.764950e−01			0.85 (0.39 − 1.84)	0.67798560			0.80 (0.37 − 1.76)	0.58726657	
Colorectal cancer	169490(2630)		1.15 (0.97 − 1.37)	1.020560e−01			1.07 (0.90 − 1.27)	0.44167447			1.04 (0.87 − 1.24)	0.65470764	
Pancreatic cancer	167430(570)		1.03 (0.70 − 1.51)	8.931438e−01			0.86 (0.58 − 1.27)	0.44981578			0.87 (0.58 − 1.29)	0.48241931	
Thyroid cancer	166948(88)		0.88 (0.32 − 2.39)	7.960670e−01			0.87 (0.31 − 2.40)	0.78109127			0.91 (0.33 − 2.53)	0.85662840	
Mouth cancer	166973(113)		1.24 (0.58 − 2.68)	5.749241e−01			0.89 (0.41 − 1.95)	0.77291326			0.84 (0.38 − 1.86)	0.66170579	
Stomach cancer	167242(382)		1.07 (0.68 − 1.70)	7.691220e−01			0.79 (0.49 − 1.26)	0.31875822			0.76 (0.47 − 1.22)	0.24970796	
Esophageal cancer	167471(611)		1.34 (0.96 − 1.87)	8.363737e−02			1.00 (0.71 − 1.41)	0.98925984			0.95 (0.67 − 1.34)	0.76887482	
Bladder cancer	167561(701)		1.06 (0.75 − 1.51)	7.292602e−01			0.91 (0.64 − 1.30)	0.61363335			0.93 (0.65 − 1.32)	0.67053428	
Leukemia	167512(652)		1.30 (0.94 − 1.80)	1.084803e−01			1.13 (0.81 − 1.57)	0.48010211			1.10 (0.79 − 1.54)	0.57665396	
Kidney cancer	167634(774)		1.18 (0.87 − 1.60)	2.953602e−01			1.04 (0.76 − 1.42)	0.81456130			0.97 (0.71 − 1.33)	0.84804504	
Eye cancer	166911(51)		1.59 (0.57 − 4.43)	3.720326e−01			1.41 (0.50 − 4.03)	0.51778728			1.42 (0.49 − 4.11)	0.52266919	
Multiple myeloma	167294(434)		1.18 (0.78 − 1.79)	4.218604e−01			1.15 (0.76 − 1.75)	0.51057297			1.15 (0.75 − 1.76)	0.51811320	
Liver cancer	167212(352)		1.84 (1.26 − 2.70)	1.644769e−03	**		1.19 (0.81 − 1.77)	0.37404368			1.06 (0.71 − 1.58)	0.77906020	
Tonsil cancer	167016(156)		0.82 (0.38 − 1.75)	6.065831e−01			0.59 (0.27 − 1.27)	0.17881597			0.62 (0.29 − 1.36)	0.23668309	
Lymphoma	167894(1034)		0.96 (0.72 − 1.29)	8.099762e−01			0.89 (0.66 − 1.20)	0.44035568			0.87 (0.64 − 1.18)	0.36632525	
Brain cancer	167234(374)		0.86 (0.52 − 1.41)	5.420643e−01			0.80 (0.48 − 1.33)	0.38792694			0.87 (0.52 − 1.45)	0.58458462	
Mesothelioma	167124(264)		0.98 (0.54 − 1.80)	9.585635e−01			0.78 (0.42 − 1.43)	0.42023118			0.76 (0.41 − 1.42)	0.39155369	
Tongue cancer	167027(167)		1.31 (0.71 − 2.42)	3.853149e−01			0.89 (0.47 − 1.67)	0.71346117			0.86 (0.46 − 1.63)	0.64663624	
Laryngeal cancer	166995(135)		2.14 (1.21 − 3.80)	9.227365e−03	**		1.09 (0.60 − 1.96)	0.77762125			1.10 (0.60 − 2.00)	0.76543903	
Biliary duct cancer	166984(124)		1.85 (0.97 − 3.53)	6.369808e−02			1.37 (0.71 − 2.67)	0.34694725			1.41 (0.72 − 2.77)	0.31555435	
Anus Cancer	166917(57)		1.43 (0.52 − 3.95)	4.939409e−01			1.10 (0.39 − 3.10)	0.86127472			1.18 (0.41 − 3.35)	0.76125791	
Small intestine cancer	166965(105)		1.20 (0.52 − 2.73)	6.685559e−01			1.19 (0.51 − 2.74)	0.68987938			1.00 (0.42 − 2.35)	0.99957571	

Panel (A) illustrates the effect of SI on specific cancer types in males using model1, model2, and model3, (B) illustrates the effect of loneliness on specific cancer types in males using model1, model2, and model3. HR and 95%CI were estimated by Cox proportional hazards model using age as the underlying time variable. The model1 was adjusted for age, sex, and race/ethnicity, the model2 was further adjusted for assessment center, employment, college/university degree, sun exposure time, socioeconomic status (Townsend deprivation score), smoking status, and alcohol use, the model3 was further adjusted for BMI (continuous), grip strength, family history of cancer, MAP, overall health rating, healthy diet score, healthy sleep score, and depressive mood. Psig was determined based on P value, with * for P < 0.05, ** for P < 0.01, *** for P < 0.001. Abbreviation: HR, Hazard Ratio; CI Confidence Intervals; BMI, Body Mass Index.

eFigure16. Associations of SI, Loneliness with Cancer Risk in Female Participants by Cancer Types (Sensitive Analysis).

A

Outcome Type	Total(Exposed)	Model1	HR(95%CI)	Pvalue	Psig	Model2	HR(95%CI)	Pvalue	Psig	Model3	HR(95%CI)	Pvalue	Psig
Melanoma	212710(1187)		0.70 (0.53 − 0.92)	1.222756e−02	*		0.79 (0.59 − 1.05)	0.1090364467			0.80 (0.60 − 1.07)	0.135963148	
Breast cancer	219111(7588)		1.18 (1.08 − 1.29)	1.729711e−04	***		1.17 (1.07 − 1.28)	0.0007910767	***		1.16 (1.06 − 1.27)	0.001366427	**
Lung cancer	213187(1664)		2.45 (2.14 − 2.80)	5.619235e−38	***		1.27 (1.10 − 1.47)	0.0009532520	***		1.22 (1.06 − 1.41)	0.007081716	**
Female genitalia cancer	211773(250)		1.08 (0.66 − 1.77)	7.505519e−01			1.01 (0.61 − 1.68)	0.9590867679			1.01 (0.61 − 1.69)	0.956168715	
Uterine cancer	212771(1248)		1.45 (1.19 − 1.76)	1.806122e−04	***		1.45 (1.19 − 1.78)	0.0002750130	***		1.37 (1.12 − 1.68)	0.002038530	**
Soft tissue cancer	211715(192)		1.54 (0.95 − 2.50)	8.245266e−02			1.39 (0.84 − 2.30)	0.1998084617			1.34 (0.81 − 2.21)	0.258490825	
Colorectal cancer	213623(2100)		1.17 (0.99 − 1.38)	6.512246e−02			1.08 (0.91 − 1.28)	0.3771021228			1.08 (0.92 − 1.29)	0.345535108	
Pancreatic cancer	212036(513)		1.42 (1.05 − 1.92)	2.449001e−02	*		1.17 (0.86 − 1.60)	0.3209574440			1.16 (0.85 − 1.59)	0.342642231	
Thyroid cancer	211769(246)		0.88 (0.50 − 1.54)	6.561088e−01			0.73 (0.41 − 1.29)	0.2767500608			0.71 (0.40 − 1.25)	0.235981580	
Mouth cancer	211591(68)		2.59 (1.32 − 5.07)	5.520957e−03	**		1.58 (0.78 − 3.23)	0.2052096561			1.60 (0.78 − 3.27)	0.197783429	
Ovarian cancer	212328(805)		1.43 (1.12 − 1.83)	4.299724e−03	**		1.34 (1.04 − 1.72)	0.0251878786	*		1.33 (1.03 − 1.72)	0.026367608	*
Stomach cancer	211715(192)		1.68 (1.06 − 2.67)	2.778526e−02	*		1.35 (0.83 − 2.18)	0.2219707471			1.32 (0.82 − 2.14)	0.257221308	
Esophageal cancer	211766(243)		1.74 (1.16 − 2.62)	7.580210e−03	**		1.49 (0.97 − 2.28)	0.0664860099			1.43 (0.93 − 2.19)	0.100734218	
Bladder cancer	211736(213)		2.45 (1.67 − 3.58)	4.126261e−06	***		1.73 (1.16 − 2.59)	0.0073557987	**		1.71 (1.14 − 2.55)	0.009047392	**
Leukemia	211966(443)		0.91 (0.61 − 1.35)	6.296561e−01			0.85 (0.57 − 1.28)	0.4413024465			0.84 (0.56 − 1.26)	0.391837634	
Kidney cancer	211977(454)		1.15 (0.81 − 1.64)	4.353537e−01			0.99 (0.69 − 1.43)	0.9543584885			0.94 (0.65 − 1.36)	0.743844998	
Eye cancer	211563(40)		2.64 (1.11 − 6.29)	2.850922e−02	*		1.86 (0.74 − 4.66)	0.1858418399			1.83 (0.73 − 4.60)	0.195936846	
Multiple myeloma	211854(331)		1.34 (0.91 − 1.98)	1.347666e−01			1.27 (0.85 − 1.89)	0.2500716590			1.26 (0.84 − 1.88)	0.265091916	
Liver cancer	211701(178)		1.53 (0.93 − 2.52)	9.679273e−02			1.29 (0.77 − 2.18)	0.3321864645			1.23 (0.73 − 2.07)	0.442271392	
Tonsil cancer	211578(55)		1.59 (0.63 − 3.99)	3.246436e−01			1.01 (0.39 − 2.63)	0.9872191671			1.01 (0.39 − 2.65)	0.976856684	
Lymphoma	212435(912)		0.93 (0.71 − 1.23)	6.197325e−01			0.85 (0.64 − 1.13)	0.2669180595			0.83 (0.63 − 1.11)	0.205891375	
Cervix Cancer	211618(95)		1.06 (0.46 − 2.43)	8.900782e−01			0.76 (0.33 − 1.78)	0.5326231209			0.80 (0.34 − 1.86)	0.596571584	
Brain cancer	211789(266)		0.77 (0.44 − 1.34)	3.505491e−01			0.81 (0.46 − 1.43)	0.4662571606			0.80 (0.45 − 1.41)	0.438000648	
Mesothelioma	211584(61)		0.73 (0.23 − 2.34)	5.984241e−01			0.52 (0.16 − 1.70)	0.2806845286			0.52 (0.16 − 1.69)	0.273343016	
Tongue cancer	211610(87)		1.33 (0.61 − 2.88)	4.708554e−01			1.11 (0.50 − 2.46)	0.8063008683			1.06 (0.47 − 2.36)	0.889653840	
Laryngeal cancer	211538(15)		2.36 (0.53 − 10.47)	2.589947e−01			1.48 (0.30 − 7.21)	0.6295171266			1.35 (0.27 − 6.70)	0.710830273	
Biliary duct cancer	211652(129)		1.33 (0.72 − 2.47)	3.642895e−01			1.10 (0.58 − 2.08)	0.7679826353			1.06 (0.56 − 2.00)	0.859361788	
Anus Cancer	211637(114)		1.16 (0.57 − 2.38)	6.845650e−01			0.94 (0.45 − 1.97)	0.8626067470			0.92 (0.44 − 1.94)	0.836667580	

0 1 2 3 4 lower risk higher risk (axis for each model)

B

Outcome Type	Total(Exposed)	Model1	HR(95%CI)	Pvalue	Psig	Model2	HR(95%CI)	Pvalue	Psig	Model3	HR(95%CI)	Pvalue	Psig
Melanoma	212710(1187)		0.66 (0.47 − 0.93)	1.802716e−02	*		0.71 (0.50 − 1.01)	0.055591196			0.76 (0.53 − 1.07)	0.118665689	
Breast cancer	219111(7588)		0.99 (0.88 − 1.11)	8.284657e−01			0.98 (0.87 − 1.09)	0.669786029			0.97 (0.86 − 1.09)	0.593815453	
Lung cancer	213187(1664)		1.80 (1.50 − 2.16)	3.486860e−10	***		1.20 (0.99 − 1.44)	0.059298872			1.11 (0.92 − 1.34)	0.296163463	
Female genitalia cancer	211773(250)		0.94 (0.50 − 1.78)	8.604200e−01			0.91 (0.48 − 1.72)	0.769840432			0.92 (0.48 − 1.75)	0.800277792	
Uterine cancer	212771(1248)		1.18 (0.92 − 1.53)	1.907523e−01			1.17 (0.91 − 1.51)	0.231277616			1.08 (0.83 − 1.40)	0.573835683	
Soft tissue cancer	211715(192)		0.86 (0.40 − 1.82)	6.850800e−01			0.84 (0.39 − 1.80)	0.655261407			0.75 (0.35 − 1.61)	0.453281637	
Colorectal cancer	213623(2100)		0.94 (0.75 − 1.17)	5.732740e−01			0.90 (0.72 − 1.12)	0.341942549			0.93 (0.75 − 1.16)	0.535520743	
Pancreatic cancer	212036(513)		1.05 (0.69 − 1.60)	8.059119e−01			0.95 (0.63 − 1.46)	0.830227094			0.96 (0.63 − 1.47)	0.854423286	
Thyroid cancer	211769(246)		1.14 (0.64 − 2.04)	6.560560e−01			1.02 (0.57 − 1.83)	0.951927314			0.97 (0.54 − 1.75)	0.916597890	
Mouth cancer	211591(68)		3.50 (1.73 − 7.05)	4.667435e−04	***		2.65 (1.29 − 5.43)	0.007906278	**		2.80 (1.34 − 5.85)	0.006118219	**
Ovarian cancer	212328(805)		1.06 (0.76 − 1.47)	7.528145e−01			1.01 (0.72 − 1.41)	0.955649492			1.01 (0.72 − 1.42)	0.940574275	
Stomach cancer	211715(192)		1.48 (0.83 − 2.66)	1.883204e−01			1.27 (0.70 − 2.29)	0.429334647			1.21 (0.66 − 2.20)	0.540933764	
Esophageal cancer	211766(243)		1.60 (0.96 − 2.65)	6.974357e−02			1.41 (0.84 − 2.35)	0.190474005			1.31 (0.78 − 2.21)	0.306124885	
Bladder cancer	211736(213)		1.71 (1.01 − 2.90)	4.440227e−02	*		1.43 (0.84 − 2.43)	0.190535868			1.46 (0.85 − 2.50)	0.167741888	
Leukemia	211966(443)		0.90 (0.56 − 1.47)	6.793862e−01			0.86 (0.53 − 1.41)	0.558605793			0.85 (0.52 − 1.39)	0.523379406	
Kidney cancer	211977(454)		1.48 (1.01 − 2.18)	4.307078e−02	*		1.34 (0.91 − 1.98)	0.135304346			1.24 (0.84 − 1.83)	0.288114092	
Eye cancer	211563(40)		1.20 (0.29 − 4.98)	7.994876e−01			1.03 (0.25 − 4.34)	0.964527191			0.94 (0.22 − 4.03)	0.928353372	
Multiple myeloma	211854(331)		1.05 (0.63 − 1.76)	8.507811e−01			1.04 (0.62 − 1.75)	0.885405661			1.05 (0.62 − 1.79)	0.842427490	
Liver cancer	211701(178)		2.09 (1.23 − 3.55)	6.323896e−03	**		1.75 (1.02 − 2.99)	0.041106587	*		1.58 (0.91 − 2.73)	0.101664012	
Tonsil cancer	211578(55)		1.31 (0.41 − 4.21)	6.461541e−01			1.01 (0.31 − 3.28)	0.989921543			1.07 (0.33 − 3.53)	0.908189119	
Lymphoma	212435(912)		1.20 (0.89 − 1.62)	2.201555e−01			1.13 (0.84 − 1.53)	0.418988166			1.05 (0.77 − 1.42)	0.768660939	
Cervix Cancer	211618(95)		1.22 (0.49 − 3.00)	6.683094e−01			1.05 (0.42 − 2.60)	0.921619584			1.24 (0.49 − 3.10)	0.649847408	
Brain cancer	211789(266)		1.36 (0.81 − 2.29)	2.500443e−01			1.35 (0.80 − 2.29)	0.259027129			1.32 (0.77 − 2.25)	0.307380160	
Mesothelioma	211584(61)		2.02 (0.81 − 5.06)	1.309220e−01			1.75 (0.70 − 4.42)	0.234288765			1.62 (0.63 − 4.16)	0.319888291	
Tongue cancer	211610(87)		1.37 (0.56 − 3.38)	4.944788e−01			1.31 (0.52 − 3.26)	0.564312990			1.19 (0.47 − 3.03)	0.713455672	
Laryngeal cancer	211538(15)		1.63 (0.21 − 12.42)	6.354054e−01			1.17 (0.15 − 9.18)	0.881143336			0.75 (0.09 − 6.46)	0.797171459	
Biliary duct cancer	211652(129)		0.91 (0.37 − 2.22)	8.300519e−01			0.81 (0.33 − 1.99)	0.644305787			0.72 (0.29 − 1.79)	0.478910851	
Anus Cancer	211637(114)		1.50 (0.70 − 3.22)	2.997596e−01			1.35 (0.62 − 2.94)	0.441786767			1.30 (0.59 − 2.85)	0.514558969	

0 1 2 3 4 lower risk higher risk (axis for each model)

Panel (A) illustrates the effect of SI on specific cancer types in females using model1, model2, and model3, (B) illustrates the effect of loneliness on specific cancer types in females using model1, model2, and model3.HR and 95%CI were estimated by Cox proportional hazards model using age as the underlying time variable. The model1 was adjusted for age, sex, and race/ethnicity, the model2 was further adjusted for assessment center, employment, college/university degree, sun exposure time, socioeconomic status (Townsend deprivation score), smoking status, and alcohol use, the model3 was further adjusted for BMI (continuous), grip strength, family history of cancer, MAP, overall health rating, healthy diet score, healthy sleep score, and depressive mood. Psig was determined based on P value, with * for P < 0.05, ** for P < 0.01, *** for P < 0.001. Abbreviation: HR, Hazard Ratio; CI Confidence Intervals; BMI, Body Mass Index; SI, Social Isolation.
