# Supplementary material for: A study of the associations between social isolation and loneliness with sex-specific cancer risk in the UK Biobank
Source: Commun Med (Lond). 2026 Mar 2;6:200. doi: 10.1038/s43856-026-01429-5 (PMC13066002; doi:10.1038/s43856-026-01429-5)
Supplement: Supplementary file 3 — Description of Additional Supplementary files [file 43856_2026_1429_MOESM3_ESM.pdf]

Description of Additional Supplementary Files

Supplementary Data 1: Linked to Figure 3

Legend: This dataset provides the Percentage of Excess Risk Mediated (PERM) by various covariate groups for the association between Social Isolation and cancer incidence. Data are presented for the Overall Population (corresponding to Figure 3A) and Female Population (corresponding to Figure 3B). The PERM values were calculated based on Cause-Specific Hazard Ratios (CSHR) and Subdistribution Hazard Ratios (sHR) from Model 3. Mediator categories include Socioeconomic Status (Townsend Deprivation Index), Health Behaviors (e.g., smoking, alcohol, sleep), Physical Health Indicators (BMI, grip strength, MAP), Mental Health (depressive mood), Overall Health Rating, Family History, and Geographical Factors. Abbreviations: SI, Social Isolation; PERM, Percentage of Excess Risk Mediated; CSHR, Cause-Specific Hazard Ratio; sHR, Subdistribution Hazard Ratio. Notes: Red font indicates statistical significance ($P < 0.05$).

Supplementary Data 2: Linked to Figure 4

Legend: This table lists the Hazard Ratios (HR) and 95% Confidence Intervals (CI) for the association of Social Isolation and Loneliness with cancer risk, stratified by demographic and lifestyle factors. The analysis assesses both multiplicative and additive interactions across subgroups including Age, Sex, Education, Employment Status, Income, Alcohol Consumption, and Smoking Status. Statistical metrics include the Number of Events, Total Participants in each stratum, and P-values for interaction. Notes: Models were adjusted for full covariates (Model 3). Red font indicates statistical significance ($P < 0.05$).

Supplementary Data 3: Linked to Figure 5

Legend: Underlying numerical data for the heatmaps illustrating the associations of Social Isolation and Loneliness with specific cancer risks across various population subgroups. The dataset includes the calculated Hazard Ratios (HR) representing the intensity of risk for each intersection of Subgroup (rows) and Cancer Outcome/Population (columns). Notes: Red font indicates statistical significance ($P < 0.05$).

Supplementary Data 4: Linked to Figure 6

Legend: Mediation analysis results for participants with Social Isolation exposure. This dataset details the mediation effects of inflammatory markers on specific cancer risks. Markers analyzed include Leukocyte count, Neutrophil count, Monocyte count, Lymphocyte count, Platelet count, C-reactive protein (CRP), and systemic indices (LMR, NLR, PLR, SII). Columns include Total Effect, Direct Effect, Indirect Effect, and Proportion Mediated (PM) with corresponding 95% Confidence Intervals. Analysis performed using the causal mediation framework. Abbreviations: LMR, Lymphocyte to Monocyte Ratio; NLR, Neutrophil to Lymphocyte Ratio; SII, Systemic Immune-Inflammation Index. Notes: Red font indicates statistical significance (P < 0.05).

Supplementary Data 5: Linked to eFigure3

Legend: Cumulative Incidence Function (CIF) and Kaplan-Meier (KM) curve data points for cancer incidence stratified by Social Isolation and Loneliness status. The dataset accounts for non-cancer death as a competing risk over a follow-up period of up to 14 years. Columns include Time (years), Cumulative Incidence Rate (%), and Standard Error for each exposure group (Isolated vs. Non-Isolated; Lonely vs. Non-Lonely).

Supplementary Data 6: Linked to eFigure4

Legend: This dataset presents the Percentage of Excess Risk Mediated (PERM) by covariates for the associations that were secondary or non-significant in the main analysis. Data are stratified by sex and overall population, detailing the contribution of Socioeconomic, Behavioral, and Health-related factors to the observed Hazard Ratios. Notes: Red font indicates statistical significance (P < 0.05).

Supplementary Data 7: Linked to eFigure5

Legend: Mediation analysis data assessing the role of Hormone and Menopause Status on specific cancer risks in Female participants exposed to Social Isolation. Mediators include reproductive factors. The table lists the Total, Direct, and Indirect Effects along with the proportion of risk mediated. Notes: Red font indicates statistical significance (P < 0.05).

Supplementary Data 8: Linked to eFigure10

Legend: Sensitivity analysis results for cancer risk associated with Social Isolation and Loneliness, stratified by demographic and lifestyle factors. This dataset replicates the structure of Figure 4 but applies sensitivity constraints to test the robustness of the

findings against reverse causality. Notes: Red font indicates statistical significance ($P < 0.05$).

Supplementary Data 9: Linked to eFigure14

Legend: Data points for the Log-Log Plots [log(-log(survival)) vs. log(time)] used to verify the Proportional Hazards (PH) assumption for the Cox models. The dataset contains the plotted values for Social Isolation and Loneliness groups across the follow-up time. Parallel lines in the plot (derived from these data) indicate that the PH assumption holds.
